# Corynebacterium pseudodiphtheriticum Exploits Staphylococcus aureus Virulence Components in a Novel Polymicrobial Defense Strategy

**DOI:** 10.1128/mBio.02491-18

**Published:** 2019-01-08

**Authors:** Britney L. Hardy, Seth W. Dickey, Roger D. Plaut, Daniel P. Riggins, Scott Stibitz, Michael Otto, D. Scott Merrell

**Affiliations:** a F. Edward Hébert School of Medicine, Department of Microbiology and Immunology, Uniformed Services University of the Health Sciences, Bethesda, Maryland, USA; b Pathogen Molecular Genetics Section, Laboratory of Bacteriology, National Institute of Allergy and Infectious Disease, National Institutes of Health, Bethesda, Maryland, USA; c Division of Bacterial, Parasitic, and Allergenic Products, Center for Biologics Evaluation and Research, Food and Drug Administration, Silver Spring, Maryland, USA; Fred Hutchinson Cancer Research Center

**Keywords:** commensal bacteria, *Corynebacterium*, nasal microbiota, polymicrobial interactions, *Staphylococcus aureus*

## Abstract

Commensal bacteria in the human nasal cavity are known to suppress opportunistic pathogen colonization by competing for limited space and nutrients. It has become increasingly apparent that some commensal bacteria also produce toxic compounds that directly inhibit or kill incoming competitors. Numerous studies suggest that microbial species-specific interactions can affect human nasal colonization by the opportunistic pathogen Staphylococcus aureus. However, the complex and dynamic molecular interactions that mediate these effects on S. aureus nasal colonization are often difficult to study and remain poorly understood. Here, we show that Corynebacterium pseudodiphtheriticum, a common member of the normal nasal microbiota, mediates contact-independent bactericidal activity against S. aureus, including methicillin-resistant S. aureus (MRSA). Bacterial interaction assays revealed that S. aureus isolates that were spontaneously resistant to C. pseudodiphtheriticum killing could be recovered at a low frequency. To better understand the pathways associated with killing and resistance, a S. aureus transposon mutant library was utilized to select for resistant mutant strains. We found that insertional inactivation of *agrC*, which codes for the sensor kinase of the Agr quorum sensing (Agr QS) system that regulates expression of many virulence factors in S. aureus, conferred resistance to killing. Analysis of the spontaneously resistant S. aureus isolates revealed that each showed decreased expression of the Agr QS components. Targeted analysis of pathways regulated by Agr QS revealed that loss of the phenol-soluble modulins (PSMs), which are effectors of Agr QS, also conferred resistance to bactericidal activity. Transmission electron microscopy analysis revealed that C. pseudodiphtheriticum induced dramatic changes to S. aureus cell surface morphology that likely resulted in cell lysis. Taken together, these data suggest that C. pseudodiphtheriticum-mediated killing of S. aureus requires S. aureus virulence components. While S. aureus can overcome targeted killing, this occurs at the cost of attenuated virulence; loss of Agr QS activity would phenotypically resemble a S. aureus commensal state that would be unlikely to be associated with disease. Commensal competition resulting in dampened virulence of the competitor may represent an exciting and unexplored possibility for development of novel antimicrobial compounds.

## Abstract (precis)

While some individuals are nasally colonized with S. aureus, the underlying factors that determine colonization are not understood. There is increasing evidence that indicates that resident bacteria play a role; some commensal species can eradicate S. aureus from the nasal cavity. Among these, Corynebacterium pseudodiphtheriticum can eliminate S. aureus from the human nose. We sought to understand this phenomenon at a molecular level and found that C. pseudodiphtheriticum produces a factor(s) that specifically kills S. aureus. While resistant S. aureus isolates were recovered at a low frequency, resistance came at the cost of attenuated virulence in these strains. Molecular dissection of the specific strategies used by C. pseudodiphtheriticum to kill S. aureus could lead to the development of novel treatments or therapies. Furthermore, commensal competition that requires virulence components of the competitor may represent an exciting and unexplored possibility for development of novel antimicrobial compounds.

## INTRODUCTION

Staphylococcus aureus is both a commensal bacterium and a versatile opportunistic pathogen that is the causative agent of numerous types of disease, including skin and soft tissue infections (SSTIs), systemic infections, and toxin-mediated diseases (1). While typically not life-threatening, SSTIs are by far the most common S. aureus-mediated disease presentation worldwide (2, 3). Moreover, annual health care costs associated with S. aureus-mediated disease approach $14 billion in the United States alone (4, 5). To further complicate matters, many circulating S. aureus strains are highly resistant to multiple antibiotics, which compromises treatment. In turn, the lack of suitable treatment leads to more severe illness and higher mortality rates (6). Indeed, in the United States methicillin-resistant S. aureus (MRSA) infections were responsible for nearly 10,000 deaths from 2005 to 2013, representing more deaths than those caused by HIV and tuberculosis combined (7). Clearly, hospital-acquired MRSA and, increasingly, community-acquired MRSA infections are serious public health concerns (8). In fact, due to the high prevalence of circulating multidrug-resistant S. aureus strains, the World Health Organization and the Centers for Disease Control and Prevention have designated MRSA and vancomycin-resistant S. aureus as “high” and “serious” threats, respectively (9, 10).

While S. aureus can cause significant morbidity and mortality, this bacterium is a part of the nasal microbiota of approximately one-third of the population; thus, S. aureus is considered to be a commensal bacterium in these individuals (11, 12). However, asymptomatic S. aureus nasal carriage is a known risk factor for subsequent S. aureus-mediated disease. In fact, patients with invasive disease are often infected with their commensal strain (13). For example, in patients diagnosed with S. aureus-induced sepsis, 82% of blood isolates were shown by multilocus sequence typing (MLST) to match nasal isolates from the same patient (14). Thus, transmission of S. aureus from the nasal cavity to other vulnerable anatomical locations likely precedes more serious disease. Additionally, these S. aureus carriers likely serve as a reservoir for transmission of the bacterium to other individuals (14). As a result of this, the decolonization of persistent and intermittent S. aureus carriers, especially in vulnerable populations (i.e., military personnel and individuals in long-term-care facilities), has been proposed as one approach that could significantly reduce S. aureus transmission and incidence of disease (15, 16).

S. aureus-mediated disease has been extensively studied, yet the specific factors that promote S. aureus commensalism are not well understood. However, it is clear that S. aureus expresses an expansive set of virulence and colonization factors that are needed to establish itself within the host’s nasal niche. These factors include adhesin molecules that promote attachment to host nasal epithelial cells, as well as pore-forming toxins and other proteins that help the bacterium to evade immune clearance (17, 18). The expression of many of the genes that encode these factors is regulated by the well-characterized accessory gene regulator quorum sensing (Agr QS) system (1). The Agr QS system is encoded ubiquitously among S. aureus strains by the *agrBDCA* operon (19). The various genes in this operon encode AgrB, a hydrolase that processes AgrD, releasing the autoinducing peptide (AIP); AgrD, the propeptide precursor of AIP; AgrC, a sensor kinase; and the corresponding response regulator, AgrA. When cell density is high, AIP binds to AgrC, and a phosphorelay cascade is initiated that culminates with phosphorylation and activation of AgrA (19). Activated AgrA positively autoregulates the expression of *agrBDCA* as well as the important effector regulatory molecule, RNAIII. RNAIII and, in some instances, AgrA modulate expression of several S. aureus virulence and colonization genes (20, 21). Included among the list of activated genes are six *psm* genes: four *psm*α and two *psm*β genes. These genes encode the phenol-soluble modulins (PSMs), small cyclic protein toxins which are important for S. aureus virulence and are widely synthesized across strains (22). In the United States, the predominant S. aureus disease-causing genetic lineage, USA300, typically shows high levels of *agrBDCA* transcription (23). Therefore, it is hypothesized that elevated Agr QS activity directly influences S. aureus virulence (23). This hypothesis is in keeping with the fact that USA300 causes severe disease in both immunocompromised and healthy individuals (24). Therefore, not only are many S. aureus isolates resistant to multiple antibiotics, but many also show high levels of expression of the Agr QS-regulated virulence factors that mediate severe disease. The importance of the Agr QS system in modulation of the S. aureus pathogenic/commensal state is further evidenced by the fact that S. aureus isolates that are recovered after invasive infection are typically Agr QS defective (25, 26); this fact suggests that there is a selective pressure against the expression of virulence factors following invasive infection, as the bacterium continues to exist within these sites (27). Furthermore, constitutive RNAIII/*agrBDCA* expression results in reduced nasal colonization in a cotton rat model of S. aureus nasal colonization (28). Thus, it has been hypothesized that the reduction or complete loss of *agrBDCA*/RNAIII expression or genes activated by these systems represents a fundamental shift from the pathogenic state to a commensal one (29, 30).

The nasal cavity is a nutrient-poor, high-salinity niche where bacteria must compete for limited resources. This microbial competition may be considered a type of bacterial “warfare” whereby various species utilize a variety of tactics to overcome their competitors. Mounting evidence suggests that S. aureus nasal colonization is heavily dependent on intricate molecular interactions with the resident nasal microbiota (31). For example, several high-throughput sequencing analyses of the composition of the nasal microbiota have revealed an inverse relationship between the presence of the *Firmicutes* and *Actinobacteria* phyla; i.e., individuals who are colonized with a high abundance of *Actinobacteria* generally have a low abundance of *Firmicutes*, including S. aureus (32). More strikingly, the presence of the *Corynebacterium* genus alone appears to impact *Staphylococcus* nasal colonization (33). In our prior studies that examined the nasal microbiota of U.S. military recruits, we found that individuals who were colonized with *Corynebacterium* species were less likely to be colonized with *Staphylococcus,* particularly S. aureus (2, 3). Similar results have been seen in other cohorts; Yan et al. found that individuals who were colonized with a single *Corynebacterium* species, C. pseudodiphtheriticum, had a very low probability of being colonized with S. aureus (31). Moreover, they found that coculture of C. pseudodiphtheriticum and S. aureus on agar plates resulted in visible inhibition of S. aureus (31).

Though few publications have looked mechanistically at the polymicrobial interactions between S. aureus and *Corynebacterium* species, some studies do suggest that the Agr QS system may impact the interaction of S. aureus and *Corynebacterium* species. There are four possible *agr* alleles (I to IV) that are differentiated by nucleotide changes in the hypervariable region within *agrD* and *agrC*. Lina et al. utilized multiple logistic regression analysis to show that the probability of isolating S. aureus strains bearing *agr* allele I or II from the nasal cavity is extremely low in the presence of *Corynebacterium* (34). Interestingly, no such correlation was seen with S. aureus strains bearing *agr* types III and IV. Thus, *agr* allele type may influence the ability of S. aureus to coexist with *Corynebacterium* in the nasal cavity (34).

Given our lack of understanding of the molecular details that govern the interaction between C. pseudodiphtheriticum and S. aureus, we set out to characterize this process. Here, we show that C. pseudodiphtheriticum mediates contact-independent bactericidal activity against S. aureus, including MRSA. S. aureus strains bearing mutations in components of the Agr QS or showing decreased expression of the Agr QS system were able to survive exposure to C. pseudodiphtheriticum killing activity. Absence of the PSMs also conferred resistance to killing activity, independently of Agr QS activity. Thus, C. pseudodiphtheriticum directly kills S. aureus; in turn, S. aureus can indirectly resist this assault by surrendering expression of its important virulence factors, most notably the *psm* genes. Commensal competition that requires virulence components of the competitor may represent an exciting and unexplored possibility for development of novel antimicrobial compounds.

## RESULTS

### C. pseudodiphtheriticum mediates bactericidal activity against S. aureus via a contact-independent mechanism.

**FIG 1:**
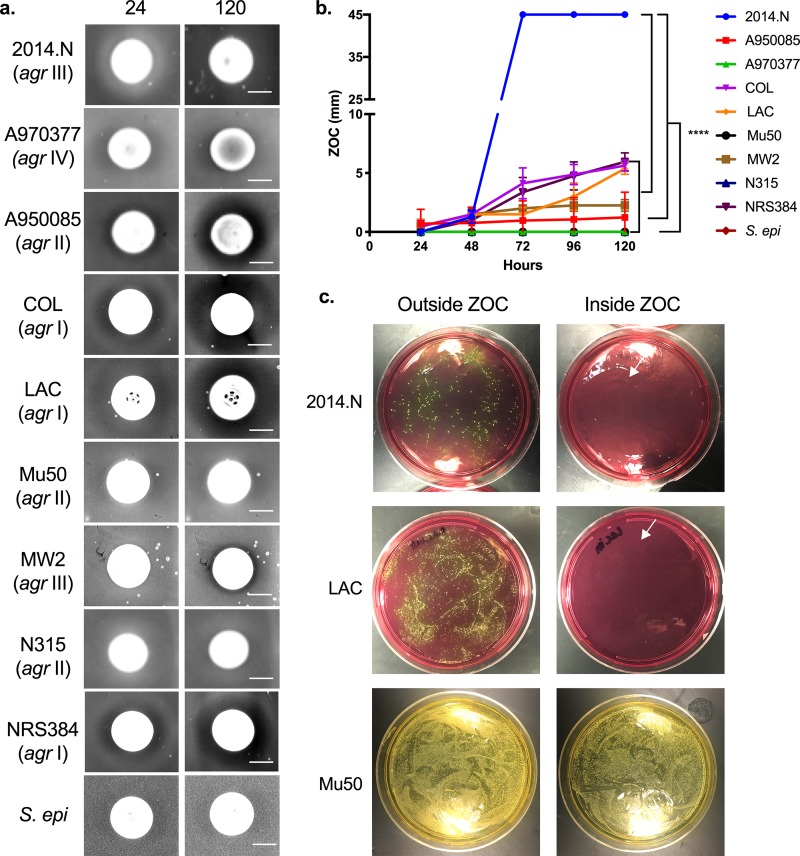
C. pseudodiphtheriticum mediates bactericidal activity against S. aureus. (a) C. pseudodiphtheriticum was spotted on agar plates that had been seeded with various S. aureus strains (2014.N, A970377, A950085, COL, LAC, Mu50, MW2, N315, and NRS384) or S. epidermidis 1457 (*S. epi*). Plates were incubated at 28°C, and images were taken every 24 h (24- and 120-hour images are shown). The *agr* allele type of each strain is given in parentheses. Bar, 10 mm. (b) The ZOC was defined as the distance between the edge of the C. pseudodiphtheriticum spot and the visible edge of the S. aureus ring of clearance. The ZOC was measured using ImageJ software (NCBI). Each symbol represents the arithmetic mean from 3 independent biological replicates measured temporally; error bars represent ±1 standard deviation. A two-way ANOVA with Dunnett’s correction for multiple comparisons was performed on the ZOC lengths at the 120-hour time point. The symbol **** on the graph indicates a statistically significant difference (*P* < 0.0001) in ZOC lengths for 2014.N versus all other strains and for Mu50 versus all other sensitive strains. (c) Punches of agar from the ZOC directly adjacent to the C. pseudodiphtheriticum or outside the ZOC were plated and grown for 48 h to recover surviving S. aureus CFU. White arrows indicate rare surviving colonies.

Data suggest that C. pseudodiphtheriticum is an important community determinant of S. aureus nasal colonization; there is an inverse relationship between nasal carriage of C. pseudodiphtheriticum and S. aureus (31). Given the lack of information concerning the reason for this negative correlation, we sought to characterize the molecular interactions of these bacterial species. We began with the prior observation that coincubation of C. pseudodiphtheriticum and S. aureus on an agar plate results in visible inhibition of S. aureus growth (31) (Fig. 1). For these initial characterization experiments, we performed modified bacterial interaction assays (31) with two strains of C. pseudodiphtheriticum: 10700, a lab-adapted strain, and USU1, a new nasal isolate recently acquired from a healthy volunteer. From these experiments, we found that C. pseudodiphtheriticum USU1 mediated a larger zone of clearance (ZOC) against S. aureus COL than C. pseudodiphtheriticum 10700 (data not shown). This result suggests that the potency of C. pseudodiphtheriticum-mediated activity against S. aureus differs among C. pseudodiphtheriticum strains. We chose to utilize the more active C. pseudodiphtheriticum strain, USU1, throughout the remainder of this study. To determine whether this inhibition occurred against multiple S. aureus strains, we tested a collection of isolates, including strains that fall within the clinically relevant USA300 pulse field type (Table 1), in the bacterial interaction assay. Furthermore, to determine whether growth inhibition was species specific, we also tested the sensitivity of Staphylococcus epidermidis and Staphylococcus saprophyticus. C. pseudodiphtheriticum-mediated inhibitory activity was temporally quantified by measuring the size of the ZOC that appeared around the C. pseudodiphtheriticum spot every 24 h for 120 h total. We observed that the ZOC steadily increased over time (Fig. 1a and b) and that this was true for both MRSA and methicillin-sensitive S. aureus (MSSA) strains. Strain sensitivity fell into three categories: highly sensitive, moderately sensitive, and resistant. S. aureus 2014.N, an uncharacterized recently acquired nasal isolate, was highly sensitive to C. pseudodiphtheriticum-mediated inhibition; the entire agar plate was completely cleared after 72 h of incubation. In comparison, S. aureus strains A950085, COL, LAC, MW2, and NRS384 were moderately sensitive (ZOC range of 2 to 10 mm) and S. aureus strains A970377, Mu50, and N315 were completely resistant (Fig. 1a and b). In addition, S. epidermidis and S. saprophyticus were resistant to C. pseudodiphtheriticum-mediated inhibition (Fig. 1a and data not shown). To determine whether other species within the *Corynebacterium* genus could also inhibit S. aureus in this bacterial interaction assay, we also assessed the ability of Corynebacterium accolens and Corynebacterium diphtheriae to inhibit S. aureus 2014.N, which was the most sensitive of the tested strains. No ZOC was observed with either of these other *Corynebacterium* species (data not shown). Taken together, these results suggested that C. pseudodiphtheriticum was able to specifically inhibit many, but not all, S. aureus strains via inhibition of S. aureus growth or by killing and lysing the S. aureus cells; the latter seemed the more plausible, since a lawn of S. aureus was visible at early time points, and the size of the ZOC increased with time.

**TABLE 1:** Strains used in this study^*a*^

Strain	Lab strain designation	Origin	Yr isolated	Notes	Reference(s)
C. pseudodiphtheriticum USU1	DSM1434	Nose	2014	Commensal	This study
C. pseudodiphtheriticum ATCC 10700	DSM1447	Throat	1980s	Commensal	72
C. accolens ATCC 49725	DSM1448	Cervix	1991	Commensal	73
C. diphtheriae	DSM1380	—	—	Commensal	This study
S. aureus COL	DSM1450	Nose	1961	Early MRSA isolate	74
S. aureus NRS384	DSM1471	Abscess	Early 1990s	CA-MRSA, USA300	—
S. aureus NRS384 transposon library	DSM1470	—	2015	Mariner transposon library	This study
S. aureus LAC	DSM1485	Blood	2005	CA-MRSA	75
S. aureus JE2	DSM1513	—	—	S. aureus LAC derivative	37, 76
S. aureus MW2	DSM1483	Blood	1998	CA-MRSA	77
S. aureus 2014.N	DSM1416	Nose	2012	Commensal	This study
S. aureus 2014.N Survivor A	DSM1652	—	2015	Spontaneously resistant isolate	This study
S. aureus 2014.N Survivor B	DSM1653	—	2015	Spontaneously resistant isolate	This study
S. aureus N315	DSM1634	Throat	1982	HA-MRSA	78
S. aureus Mu50	DSM1633	Abscess	1997	HA-MRSA	78
S. aureus A950085	DSM1685	—	—	Clinical isolate	79
S. aureus A970377	DSM1686	—	—	Clinical isolate	79
S. epidermidis 1457	DSM1637	**—**	—	Commensal	80
S. epidermidis 1457 pTX_Δ_*psm*α1-4	DSM1687	—	—	—	This study
S. epidermidis 1457 pTX_Δ_16	DSM1688	—	—	Empty vector control	This study
S. saprophyticus	DSM1655	Urine	—	—	This study
S. aureus JE2 *Tn*::*agrA*	DSM1640	—	—	—	37
S. aureus JE2 *Tn*::*agrB*	DSM1641	—	—	—	37
S. aureus JE2 *Tn*::*agrC*	DSM1639		—	—	37
S. aureus JE2 *Tn*::*agrC* pCL15-P_spac_-*agrC*	DSM1654	—	This study	Complemented strain	This study
S. aureus JE2 *Tn*::*agrC* pTX_Δ_*psm*α1-4	DSM1689	—	This study	pTX_Δ_*psm*α1-4, carries α-*psm* genes	This study
S. aureus LAC Δ*agrBDCA*	DSM1486	—	2013	Transduction of *agr* deletion from RN6911, Tet^r^	23
S. aureus MW2 Δ*agrBDCA*	DSM1484	—	2013	Transduction of *agr* deletion from RN6911, Tet^r^	23
S. aureus LAC Δ*psm*	DSM1520	—	2012	Deletions of *psm*β and *psm*α operons, mutation of start codon of *hld* to nonsense, Spec^r^	39
S. aureus LAC *Δpsm* pTX_Δ_*psm*α1-4	DSM1523	—	2014	Complemented strain	39

a Symbols and abbreviations: —, specific information was unavailable or not applicable. CA, community acquired; HA, hospital acquired.

To our knowledge, there is no published evidence that any *Corynebacterium* species possesses bactericidal activity against S. aureus. Thus, to determine if C. pseudodiphtheriticum-mediated inhibition was due to S. aureus growth inhibition (bacteriostatic) or due to killing of the S. aureus cells (bactericidal), we attempted to recover surviving S. aureus directly from the ZOC. Agar punches were taken from inside the ZOC for highly sensitive (2014.N), intermediate sensitive (LAC) and resistant (Mu50) strains; we reasoned that even though a ZOC did not form around a resistant strain, the S. aureus cells might still be dead within that region. Similar punches were taken from outside the ZOC for qualitative comparison and to estimate the frequency of any spontaneous resistance. While large numbers of bacteria were recovered from outside the ZOC, only rarely were colonies recovered from inside the ZOC for strain 2014.N or LAC. Conversely, similar numbers of colonies were recovered from outside and within the ZOC for Mu50, a resistant isolate (Fig. 1c). Since some colonies were recovered from the ZOC of sensitive strains, we hypothesized that these bacteria could represent spontaneously resistant isolates. To test this, we randomly selected five recovered 2014.N isolates and assayed them individually for sensitivity to C. pseudodiphtheriticum-mediated inhibition. All five isolates (Survivors A to E) were completely resistant to inhibition (see Fig. S1 in the supplemental material), as no ZOC formed. Thus, sensitive S. aureus strains can develop spontaneous resistance to C. pseudodiphtheriticum-mediated inhibition. Using the number of bacterial colonies obtained from outside and within the ZOC for both 2014.N and LAC, we estimated that the frequency of spontaneous resistance was approximately 10^−8^. *En masse*, these data indicate that C. pseudodiphtheriticum selectively mediates bactericidal activity against many S. aureus strains and that spontaneous resistance to this activity occurs at a low frequency.

10.1128/mBio.02491-18.2 FIG S1 Isolates recovered from the ZOC are stably resistant to C. pseudodiphtheriticum bactericidal activity. Approximately 10^9^ C. pseudodiphtheriticum cells were spotted on a BHIT agar plate seeded with ∼10^8^ cells of 2014.N. Punches of agar from the ZOC were taken at 48 hours and plated on MSA. Five surviving isolates were chosen at random (Survivors A to E) and reassayed for sensitivity. Images were taken after 120 hours of incubation at 28°C and are representative of three independent biological replicates. Download FIG S1, TIF file, 2.5 MB. Copyright © 2019 Hardy et al. 2019 Hardy et al. This content is distributed under the terms of the Creative Commons Attribution 4.0 International license.

**FIG 2:**
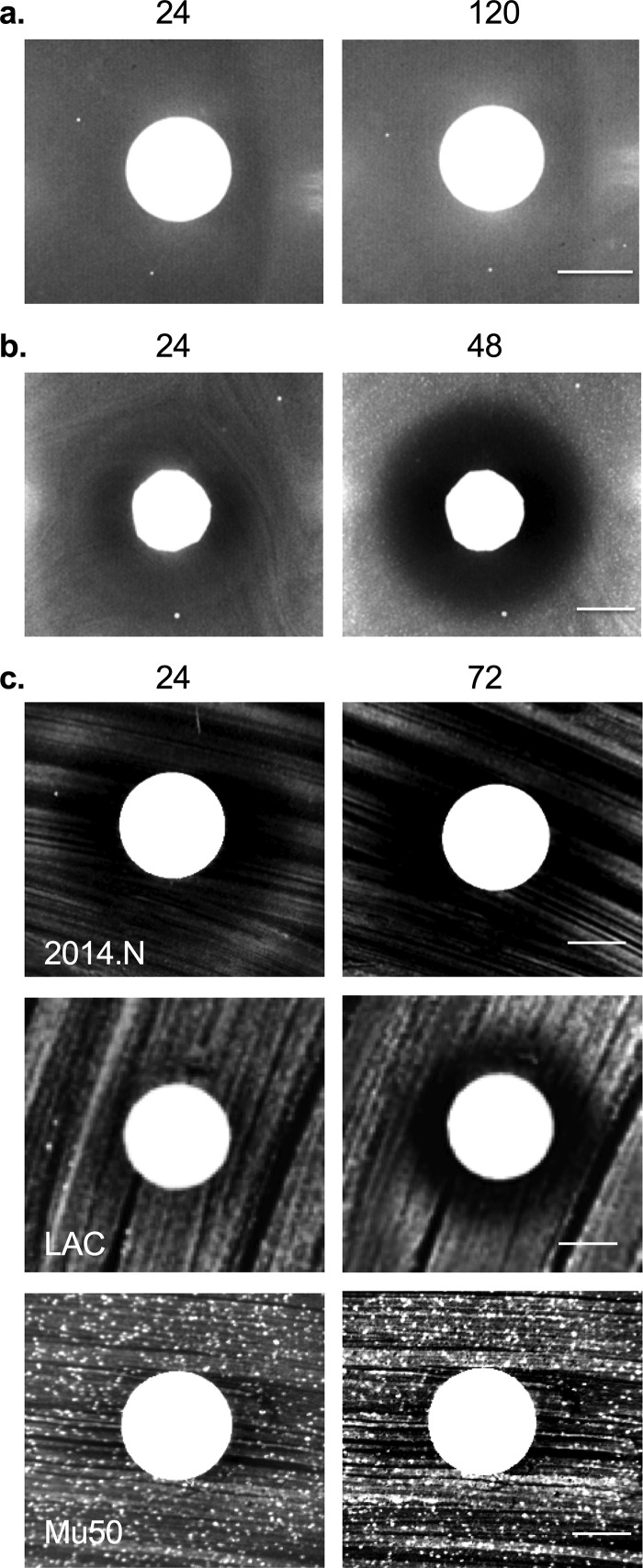
Bactericidal activity against S. aureus requires live C. pseudodiphtheriticum and is contact independent. (a) C. pseudodiphtheriticum cells were heated to 90°C for 10 min and then spotted on BHIT agar seeded with S. aureus 2014.N. (b) A 0.2-μm filter was placed on top of BHIT agar seeded with S. aureus 2014.N, and C. pseudodiphtheriticum was spotted on top of the filter. Images of the ZOC were taken after 24, 48, and 120 h of incubation at 28°C; 24- and 120-hour images are shown in panel a, and 24- and 48-hour images are shown in panel b. Images are representative of three independent biological replicates. Bar, 10 mm. (c) S. aureus strains 2014.N, LAC, and Mu50 were spread on the agar surface, and a sterile disk was placed in the center of the plate. Fifty microliters of concentrated CCFM prepared from C. pseudodiphtheriticum was inoculated onto the disk and allowed to dry. Images of the ZOC were taken after 24 and 72 h of incubation. Images are representative of three independent biological replicates. Bar, 10 mm.

In order to further characterize this interaction at a molecular level, we next performed two variations of the bacterial interaction assay. To determine whether C. pseudodiphtheriticum-mediated bactericidal activity required live bacteria, heat-killed C. pseudodiphtheriticum cells were utilized; no ZOC was formed (Fig. 2a). To determine whether bactericidal activity was dependent on direct contact, C. pseudodiphtheriticum and S. aureus cells were separated with a permeable 0.2-μm filter disk; the ZOC formed as before (Fig. 2b). Given this indication that bactericidal activity was mediated by a secreted factor(s), we next asked whether this factor(s) would be present in conditioned cell-free medium (CCFM) in which C. pseudodiphtheriticum had been grown overnight. Activity of concentrated CCFM was tested via disk diffusion assays against S. aureus strains that fell into the various C. pseudodiphtheriticum sensitivity categories: 2014.N (highly sensitive), LAC (moderately sensitive), and Mu50 (resistant). Mimicking the degree of bactericidal activity mediated by live C. pseudodiphtheriticum (Fig. 1a), a visible ZOC formed with 2014.N and LAC but not with Mu50 (Fig. 2c). Overall, these data indicate that C. pseudodiphtheriticum-mediated bactericidal activity is an active process that is mediated by a secreted factor(s).

### Identification of potential target(s) of bactericidal activity using a S. aureus Tn mutant library.

**FIG 3:**
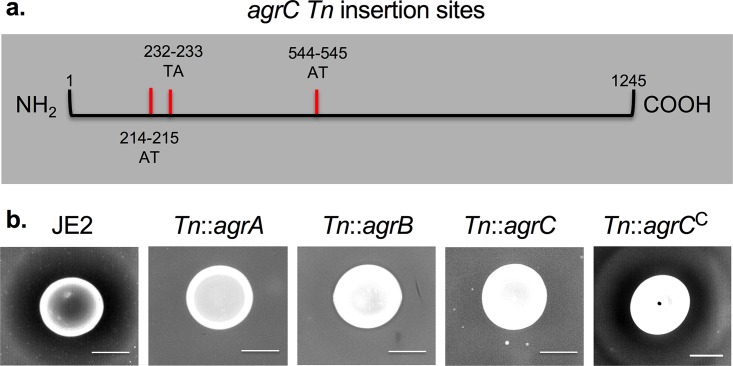
Absence of *agrBDCA* confers resistance to C. pseudodiphtheriticum-mediated bactericidal activity. (a) *agrC* insertion sites were mapped against the NCTC 8325 S. aureus reference genome (NCBI). The sites of insertion are indicated as hatch marks, and the nucleotide position (number) and bases flanking the transposon insertion site (letters) are shown. (b) S. aureus JE2 and JE2 strains containing Tn insertions in *agrA*, *agrB*, *agrC*, and *agrC*^C^ were tested for sensitivity to C. pseudodiphtheriticum-mediated bactericidal activity. Images of the ZOC were taken after 120 h of incubation at 28°C and are representative of three independent biological replicates. Bar, 10 mm.

Given that we observed spontaneous resistance of S. aureus sensitive strains to C. pseudodiphtheriticum-mediated bactericidal activity at a relatively low frequency (10^−8^), we reasoned that we should be able to use a S. aureus transposon (Tn) library to select for surviving transposants. Therefore, we repeated the bacterial interaction assay using a pooled S. aureus NRS384 *H1 mariner* transposon library; we recovered resistant transposant isolates from the ZOC. The experiment was independently repeated three times and yielded a total of 31 recovered resistant transposon isolates. The site of transposon insertion was identified using a rescue cloning strategy and revealed insertions within 6 separate genes (Table 2). The most frequently recovered transposants (14/31) had insertions in *agrC*, which encodes AgrC, the sensor kinase that is a part of the Agr QS system (Table 2). These 14 recovered isolates represented three independent transposon insertion events within the *agrC* open reading frame (Fig. 3a), strongly suggesting that disruption of *agrC* reduced sensitivity to C. pseudodiphtheriticum-mediated bactericidal activity. The remaining recovered resistant transposon mutants had insertions in genes predicted to be involved in *de novo* nucleotide synthesis (*carA*, *purL*, *purK*, and *pyrF*) (35) and peptidoglycan synthesis (*bacA*) (36) (Table 2). A representative isolate bearing a transposon insertion in each of the six genes was independently retested in the bacterial interaction assay and was confirmed to be resistant to C. pseudodiphtheriticum-mediated bactericidal activity (Fig. S2). Given the importance of the Agr QS system for S. aureus virulence and because previous studies have demonstrated that some *Corynebacterium* species, including C. pseudodiphtheriticum, can induce changes in S. aureus Agr QS activity (36), we focused the remainder of our studies on gaining a more thorough understanding of the role of Agr QS in the context of C. pseudodiphtheriticum-mediated bactericidal activity.

**TABLE 2:** Recovered resistant transposon mutant strains

Gene	No. of colonies recovered	Location	Pathway	No. of independent insertion sites
*agrC*	14	Chromosome	Quorum sensing	3
*bacA*	5	Plasmid	Peptidoglycan synthesis	2
*carA*	3	Chromosome	*De novo* pyrimidine synthesis	1
*purL*	4	Chromosome	*De novo* purine synthesis	1
*purK*	3	Chromosome	*De novo* purine synthesis	1
*pyrF*	2	Chromosome	*De novo* pyrimidine synthesis	1

10.1128/mBio.02491-18.3 FIG S2 Transposon mutant strains recovered from the ZOC are stably resistant to C. pseudodiphtheriticum bactericidal activity and have reduced α-*psm* expression. Approximately 10^9^ C. pseudodiphtheriticum cells were spotted on a BHIT agar plate seeded with ∼10^8^ cells of NRS384 or NRS384 mutant strains that contained Tn insertions in *agrC*, *bacA*, *carA*, *purK*, *purL*, or *pyrF.* Images were taken after 120 hours of incubation at 28°C and are representative of three independent biological replicates. Bar, 10 mm. α-*psm* gene expression is shown relative to 16S rRNA gene expression for three independent biological replicates. The data were plotted as follows: the geometric mean is represented as a bar, and error bars represent ±1 geometric standard deviation. A two-way ANOVA with Dunnett’s corrections for multiple comparisons was performed. The symbol *** above the graph indicates statistically significant difference (*P* ≤ 0.001) α-*psm* gene expression for NRS384 versus *agrC*, *carA*, *purK*, *purL*, and *pyrF* mutant strains. Download FIG S2, TIF file, 2.3 MB. Copyright © 2019 Hardy et al. 2019 Hardy et al. This content is distributed under the terms of the Creative Commons Attribution 4.0 International license.

### Absence of or decreased expression of *agrBDCA* confers resistance to C. pseudodiphtheriticum-mediated bactericidal activity.

The NRS384 transposon mutant screen described above revealed that insertional inactivation of *agrC* conferred resistance to C. pseudodiphtheriticum-mediated bactericidal activity. However, we noted that we did not recover resistant mutant isolates that had insertions in the other genes of the *agrBDCA* operon. The NRS384 *H1 mariner* pooled transposon library that was initially used was not fully characterized at the time of this study; thus, transposants with insertions in *agrA*, *agrB*, and *agrD* may not exist within the transposon pool. As expression of *agrBDCA* is autoregulated by AgrA, we hypothesized that insertional inactivation of any gene in the *agrBDCA* operon should confer resistance to C. pseudodiphtheriticum-mediated bactericidal activity. To test this, we analyzed the sensitivity of S. aureus JE2 (USA300, LAC derivative) and JE2 transposon mutant strains that contained Tn insertions in *agrB*, *agrA*, and *agrC* to C. pseudodiphtheriticum-mediated bactericidal activity (Fig. 3b). We observed that insertional inactivation of any of these genes conferred resistance to bactericidal activity. Furthermore, this phenotype was complementable; complementation of *agrC* in the *Tn*::*agrC* mutant strain (*Tn*::*agrC*^C^) restored sensitivity to bactericidal activity (Fig. 3b). To further ensure that complementation of *agrC* alone restored Agr QS activity in the *Tn*::*agrC* mutant strain, hemolysis assays were also performed; red blood cell hemolysis is a crude measure of Agr QS activity (37). Indeed, hemolysis activity was restored in *Tn*::*agrC*^C^ (data not shown). To extend these results to other S. aureus strain backgrounds, we also tested the sensitivity of isogenic Δ*agrBDCA* mutant strains constructed in the MW2 and LAC strains. While each of the parental strains was sensitive to C. pseudodiphtheriticum-mediated bactericidal activity, no ZOC formed with either Δ*agrBDCA* mutant strain (Fig. S3). Thus, loss of Agr QS activity conferred resistance to C. pseudodiphtheriticum-mediated bactericidal activity across multiple S. aureus strain backgrounds.

10.1128/mBio.02491-18.4 FIG S3 Absence of *agr* expression confers resistance to C. pseudodiphtheriticum-mediated bactericidal activity. Approximately 10^9^ C. pseudodiphtheriticum cells were spotted on a BHIT agar plate seeded with ∼10^8^ cells of S. aureus LAC, LACΔ*agrBDCA*, MW2, or MW2Δ*agrBDCA.* Images of the ZOC were taken after 120 hours of incubation at 28°C and are representative of three independent biological replicates. Bar, 10 mm. Download FIG S3, TIF file, 2.6 MB. Copyright © 2019 Hardy et al. 2019 Hardy et al. This content is distributed under the terms of the Creative Commons Attribution 4.0 International license.

**FIG 4:**
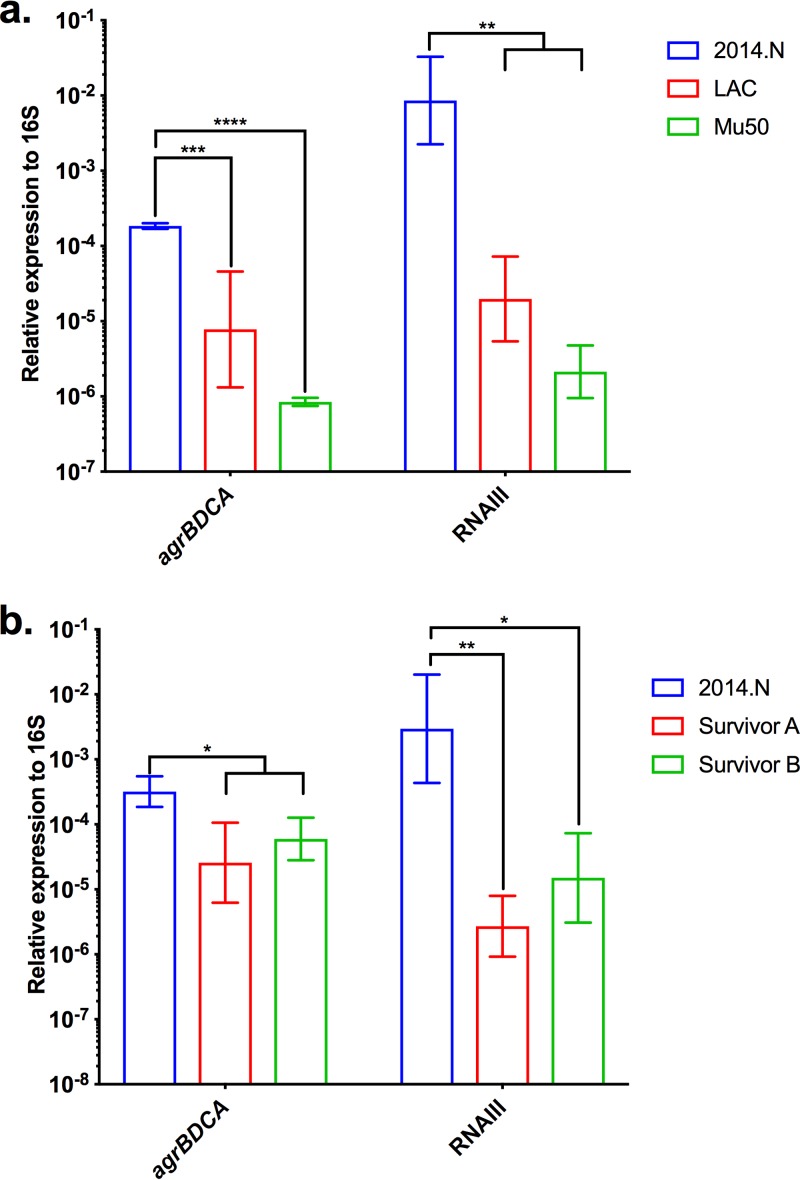
Expression of *agrBDCA* is decreased in S. aureus isolates that are resistant to C. pseudodiphtheriticum-mediated bactericidal activity. Expression of *agrBDCA* was measured by qRT-PCR using primers specific to *agrB*, the first gene in the operon. RNA was isolated from (a) S. aureus LAC, Mu50, and 2014.N and (b) two spontaneously resistant 2014.N mutant strains (Survivors A and B) after 24 h of incubation at 28°C on BHIT agar. *agrB* and RNAIII expression are shown relative to 16S rRNA gene expression for three independent biological replicates. The data were plotted as follows: the geometric mean is represented as a bar, and error bars represent ±1 geometric standard deviation. A two-way ANOVA with Dunnett’s corrections for multiple comparisons was performed. The symbols above the graph indicate the following: *, *P* < 0.05; **, *P* < 0.01; ***, *P* ≤ 0.001; and ****, *P* < 0.0001, for differences in *agrBDCA* or RNAIII gene expression for 2014.N versus the other strains.

Given that Agr QS conferred sensitivity to C. pseudodiphtheriticum-mediated bactericidal activity, we next wondered if the level of sensitivity of the various S. aureus strains (Fig. 1) would correlate with the level of expression of *agrBDCA* transcript found in these strains. Therefore, we measured *agrBDCA* expression in S. aureus strains 2014.N (highly sensitive), LAC (moderately sensitive), and Mu50 (resistant) relative to the 16S rRNA gene. The levels of *agrBDCA* expression perfectly correlated with the overall sensitivity to C. pseudodiphtheriticum-mediated killing (Fig. 4a); resistant Mu50 showed a very low level of *agrBDCA* expression, moderately sensitive LAC showed an intermediate level of expression, and highly sensitive 2014.N showed a significantly higher level of *agrBDCA* expression. Furthermore, transcript levels of RNAIII, an important effector molecule of Agr QS, mirrored the relative transcription level of *agrBDCA* (Fig. 4a). Given this, transcript levels of *agrBDCA* and RNAIII (data not shown) were also measured in strain A970377, which was also resistant to C. pseudodiphtheriticum-mediated killing activity (Fig. 1a and b). Expression of these genes was higher than in Mu50 but lower than in LAC. Thus, there may be a threshold of *agrBDCA* expression that is required for C. pseudodiphtheriticum-mediated bactericidal activity. Given these results, we next wondered if the spontaneously resistant S. aureus isolates that were recovered from the ZOC in the bacterial interaction assay (Fig. S1) showed some form of defect in *agrBDCA* or RNAIII expression. Therefore, we assessed *agrBDCA* and RNAIII transcripts in two of the spontaneously resistant 2014.N isolates that were previously recovered: Survivors A and B. Expression of *agrBDCA* and RNAIII transcript level was significantly reduced in both Survivor A and Survivor B (Fig. 4b) compared to the parental strain. These results strongly suggest that absence of or lower expression of *agrBDCA* results in protection from C. pseudodiphtheriticum-mediated bactericidal activity.

It was previously observed that S. aureus strains that carry *agr* allele I or II rarely coexist with *Corynebacterium* species in the nasal cavity (38). This observation, combined with the variability in the degree of sensitivity seen with the various strains (Fig. 1a and b), led us to ask whether the *agr* allele type of those strains corresponded to the overall degree of sensitivity to C. pseudodiphtheriticum-mediated bactericidal activity. All three strains carrying *agr* I alleles showed intermediate sensitivity. The three strains carrying *agr* II alleles showed either resistance (two strains) or intermediate sensitivity (one strain). The two *agr* III-containing strains showed high and intermediate sensitivity. The one available *agr* IV allele-containing strain was resistant. Overall, there appeared to be no direct correlation between C. pseudodiphtheriticum-mediated bactericidal activity and *agr* allele type. Thus, again we concluded that a threshold level of *agrBDCA*/RNAIII expression must exist to confer sensitivity to bactericidal activity, regardless of what *agr* allele type is encoded.

### Loss of *psm* expression confers resistance to C. pseudodiphtheriticum-mediated bactericidal activity.

**FIG 5:**
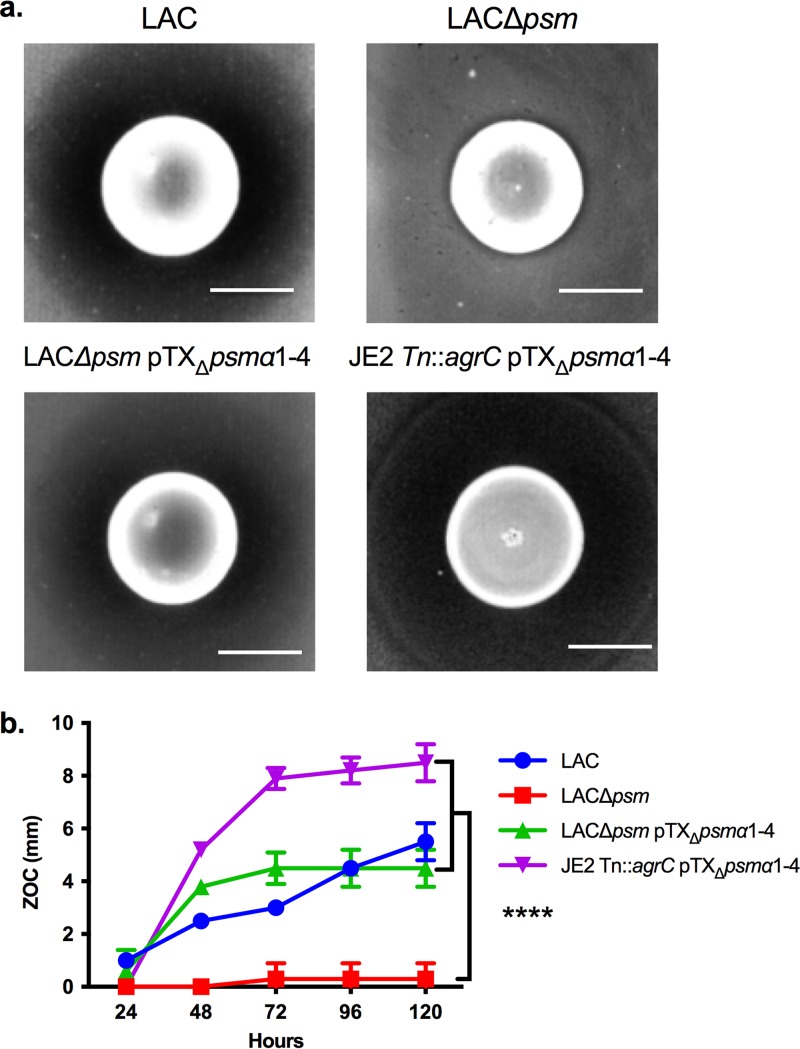
Absence of *psm* confers resistance to C. pseudodiphtheriticum-mediated bactericidal activity. (a) S. aureus LAC, LACΔ*psm*, LACΔ*psm* pTX_Δ_*psm*α1-4, and JE2 *Tn*::*agrC* pTX_Δ_*psm*α1-4 were assayed for sensitivity to C. pseudodiphtheriticum-mediated bactericidal activity. Images of the ZOC were taken after 120 h of incubation at 28°C and are representative of three independent biological replicates. Bar, 10 mm. (b) The ZOC distance was measured using ImageJ software. Each symbol represents the arithmetic mean from three independent biological replicates, and errors bars represent ±1 standard deviation. A two-way ANOVA with Dunnett’s correction for multiple comparisons was performed on the ZOC lengths at the 120-hour time point. The symbol **** on the graph indicates statistically significant differences (*P* < 0.0001) of ZOC lengths for the LACΔ*psm* strain versus all other strains.

The finding that C. pseudodiphtheriticum appears to affect S. aureus Agr QS as a mechanism to mediate bactericidal activity was unexpected given that, to our knowledge, there is no evidence that directly connects Agr QS activity to cell death. Hence, we focused on genes that are known to be regulated by Agr QS as a means to understand the link between the Agr QS system and C. pseudodiphtheriticum*-*mediated bactericidal activity. Of the known Agr-regulated factors, the PSMs were of particular interest. The PSMs are downstream effectors of Agr QS, and data indicate that aberrant buildup of the PSMs inside the S. aureus cell via disruption of the gene encoding their dedicated transporter, PMT, leads to membrane instability and eventual cell lysis (39). Thus, we questioned whether the PSMs were required for C. pseudodiphtheriticum*-*mediated bactericidal activity. To test this, we assayed the sensitivity of wild-type S. aureus LAC compared to mutant strains that contained isogenic deletions of all six *psm* genes (LAC*Δpsm*), or a complemented derivative containing the α-*psm* genes (LACΔ*psm* pTX_Δ_*psm*α1-4) under the control of a constitutively expressed promoter. The deletion of the *psm* genes resulted in resistance to C. pseudodiphtheriticum-mediated bactericidal activity, and sensitivity was restored upon complementation of the α-*psm* genes (Fig. 5a and b).

**FIG 6:**
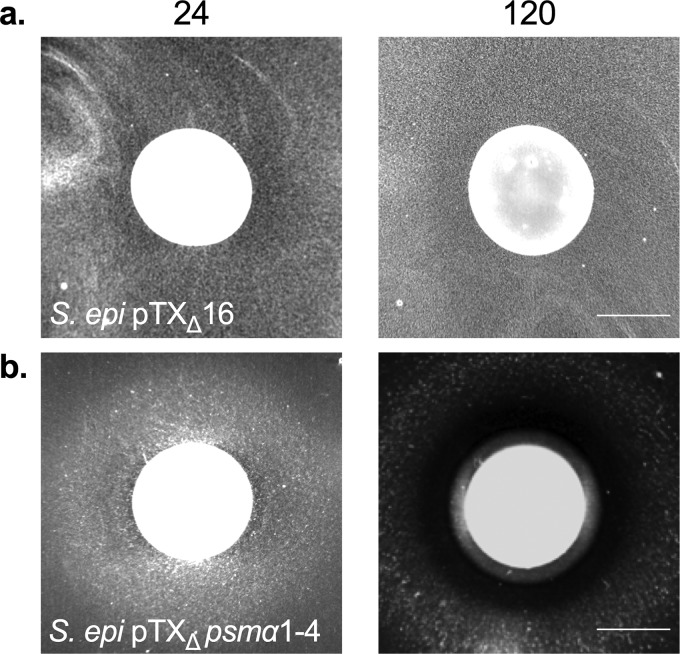
Expression of the α-*psm* genes in S. epidermidis confers sensitivity to C. pseudodiphtheriticum-mediated bactericidal activity. S. epidermidis 1457 pTX_Δ_16 (empty vector) and S. epidermidis 1457 pTX_Δ_*psm*α1-4 were assayed for sensitivity to C. pseudodiphtheriticum-mediated bactericidal activity. Images of the ZOC were taken after 24 and 120 h of incubation at 28°C and are representative of three independent biological replicates. Bar, 10 mm.

Given that *agr* mutations are pleiotropic and result in changes in expression of many genes, we next asked whether resistance of the *agr* mutant strains (Fig. 3) was specifically due to loss of expression of the α-*psm* genes. To this end, we transformed the JE2 *Tn*::*agrC* strain with the α-*psm* complementation vector (pTX_Δ_*psm*α1-4) and tested sensitivity of the strain to C. pseudodiphtheriticum-mediated bactericidal activity; the α-*psm* genes are constitutively expressed in the pTX_Δ_*psm*α1-4 vector and are not subject to Agr-mediated regulation. JE2 *Tn*::*agrC* pTX_Δ_*psm*α1-4 was fully sensitive to C. pseudodiphtheriticum-mediated bactericidal activity (Fig. 5). Furthermore, when S. epidermidis 1457, which was previously resistant to killing (Fig. 1), was transformed with the pTX_Δ_*psm*α1-4 vector, S. epidermidis 1457 pTX_Δ_*psm*α1-4 became sensitive to C. pseudodiphtheriticum-mediated killing; no ZOC was formed with the S. epidermidis pTX_Δ_16 empty vector control (Fig. 6). Thus, resistance of the *agr* mutant strains (Fig. 3) appeared not to be due to any larger pleiotropic effect, and expression of the *psm* genes in S. epidermidis was sufficient to induce susceptibility.

**FIG 7:**
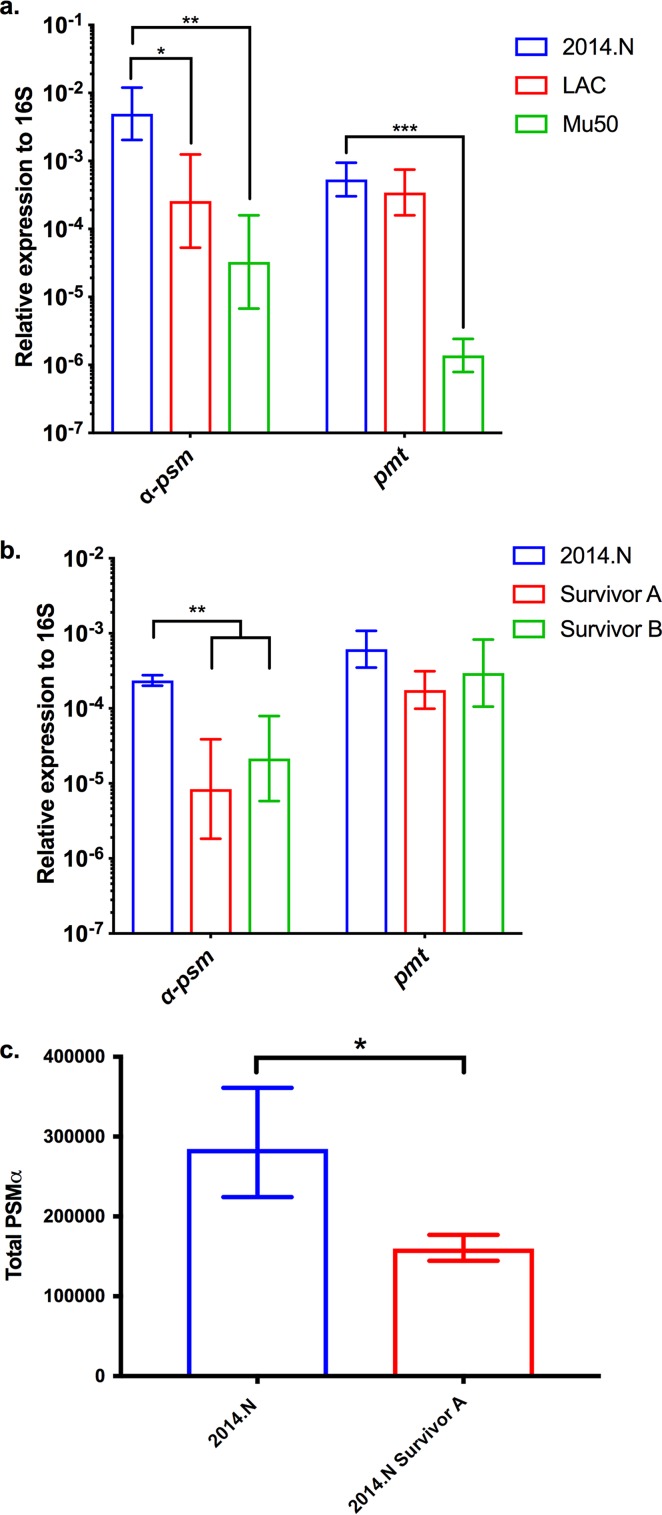
Expression of α-*psm* is decreased in S. aureus isolates resistant to C. pseudodiphtheriticum-mediated bactericidal activity. Expression of α-*psm* and *pmt* was measured by qRT-PCR using primers specific to *psm*α_1_ or *pmtA*, the first gene in each operon, respectively. (a and b) RNA was isolated from S. aureus LAC, Mu50, and 2014.N (a) and two spontaneously resistant 2014.N mutant strains (Survivors A and B) (b) after 24 h of incubation at 28°C on BHIT agar. α-*psm* and *pmt* gene expression is shown relative to 16S rRNA gene expression for three independent biological replicates. The data were plotted as follows: the geometric mean is represented as a bar, and error bars represent ±1 geometric standard deviation. A two-way ANOVA with Dunnett’s corrections for multiple comparisons was performed. The symbols above the graph indicate the following: *, *P* < 0.05; **, *P* < 0.01; and ***, *P* ≤ 0.001, for differences in α-*psm* and *pmt* gene expression for 2014.N versus the indicated strains. (c) 2014.N and 2014.N Survivor A strains were cultured overnight in BHIT broth. The total quantities of PSMs present were analyzed and measured by reverse-phase liquid chromatography–mass spectrometry. Bars represent arithmetic means from three independent biological replicates ±1 standard deviation. A two-tailed Student *t* test with Welch’s correction was performed on total PSM quantity. The symbol * above the graph indicated a statistically significant difference between 2014.N and Survivor A (*P =* 0.048).

Given the data showing the correlation between the level of *agrBDCA* expression and sensitivity to C. pseudodiphtheriticum-mediated bactericidal activity, we also assessed *psm*α_1_ (first gene in the *psm*α operon) transcript levels in S. aureus 2014.N (highly sensitive), LAC (moderately sensitive), and Mu50 (resistant) and in two of the 2014.N spontaneously resistant isolates (Survivors A and B) relative to 16S rRNA gene expression (Fig. 7). The *psm*α_1_ transcript level perfectly correlated with the overall sensitivity to C. pseudodiphtheriticum-mediated killing; resistant Mu50 showed a very low level of *psm*α_1_ expression, moderately sensitive LAC showed an intermediate level of expression, and highly sensitive 2014.N showed significantly higher *psm*α_1_ expression (Fig. 7a). Furthermore, both Survivor A and Survivor B showed significantly decreased levels of *psm*α_1_ expression (Fig. 7b) compared to 2014.N. To determine if this correlated with decreased levels of α-PSM, we measured total α-PSM concentration from parental strain 2014.N and the surviving derivative, 2014.N Survivor A. The quantity of α-PSMs produced by the spontaneously resistant isolate 2014.N Survivor A was significantly reduced compared to the parental strain (Fig. 7c). While the level of α-*psm* transcript mimicked the *agrBDCA* pattern of expression, the transcript level of *pmt* was not significantly different across strains, with the exception of 2014.N and Mu50 (Fig. 7a) (*P* = 0.0001).

Next, we asked whether defects in expression of *agrBDCA* or *psm*α_1_ could also account for the resistance seen with mutant strains containing Tn insertions in *purK*, *pyrF*, *carA*, *purK*, or *bacA*. While there was no difference in *agrBDCA* between the parental strain and any of the resistant transposon mutant strains (data not shown), differences in *psm* expression were observed. Specifically, expression of *psm*α_1_ was significantly decreased in mutant strains bearing Tn insertions in *purL*, *purK*, *pyrF*, and *carA* but not in *bacA* (Fig. S2). Therefore, defects in *psm*α expression correlate with resistance in most of the transposon mutant strains. Taken together, the expression data indicate that absence or reduction of *psm* expression confers resistance to C. pseudodiphtheriticum-mediated bactericidal activity, likely independent of Agr QS activity.

### Altered biofilm production is not responsible for resistance to C. pseudodiphtheriticum.

Both Δ*agr* and Δ*psm* mutant strains are known to have “hyper-biofilm-producing” phenotypes (40). Indeed, biofilm formation in the *agrC* mutant strain was significantly higher than in the wild-type strain, and this phenotype was lost upon transformation with pTX_Δ_*psm*α1-4 (Fig. S4a). It is well established that biofilms provide protection against antimicrobial peptides, antibiotics, and other environmental stresses (41–43). Therefore, it was plausible that the increased biofilm production of the Δ*agr* and Δ*psm* mutant strains was responsible for resistance to C. pseudodiphtheriticum-mediated bactericidal activity. To investigate this possibility, we analyzed the available literature on S. aureus biofilm production to identify other genes whose mutations result in a hyper-biofilm-producing phenotype. We took advantage of the arrayed JE2 transposon library (37) and selected transposon mutant strains that contained mutations in these genes, tested their biofilm-forming ability compared to JE2, and then tested sensitivity to C. pseudodiphtheriticum-mediated bactericidal activity. Transposon mutant strains bearing Tn insertions in *icaR* (negative regulator of biofilm production), *aur* (metalloprotease with a role in biofilm dispersal), *rsp* (represses biofilm formation), *scpA* (cysteine protease with a role in biofilm dispersal), and *splB* (cysteine protease with a role in biofilm dispersal) were all analyzed. As seen in other strain backgrounds (44–48), biofilm production was significantly increased in 2/5 selected transposon mutant strains and trended higher in the three remaining strains compared to parental JE2 (Fig. S4a and b). Moreover, though the degree of sensitivity did vary across each of the mutant strains, each of the selected hyper-biofilm-producing transposon mutant strains was still sensitive to C. pseudodiphtheriticum-mediated bactericidal killing (Fig. S4c and d). These data suggest that increased biofilm production is likely not responsible for the resistant phenotypes of the Δ*agr* and Δ*psm* mutant strains to bactericidal activity.

10.1128/mBio.02491-18.5 FIG S4 Increased biofilm production does not correlate with resistance to C. pseudodiphtheriticum-mediated bactericidal activity. (a) S. aureus biofilms were established for 48 hours in 24-well tissue culture-treated plates at 28°C. Biofilms were then washed, stained with crystal violet solution, and solubilized in differentiation solution and 100% ethanol. Solubilized crystal violet solution was read at an absorbance of 590 nm. Each symbol represents OD_590_. The data are plotted as follows: the arithmetic mean from three independent biological replicates is represented as a bar, and error bars represent ±1 standard deviation. A two-way ANOVA with Dunnett’s corrections for multiple comparisons was performed on OD_590_ values. The symbols above the graph * (*P* < 0.05) and ** (*P* < 0.01) indicate statistically different OD_590_ values for JE2 versus *splB*, *rsp*, *agrC*, and *agrC* pTX_Δ_*psm*α1-4 strains. The symbol *** (*P < *0.001) indicates a statistically significant difference in OD_590_ value for *agrC* versus *agrC* pTX_Δ_*psm*α1-4. (b) Representative images are shown of established biofilms after 48 hours of incubation at 28°C in 24-well tissue culture-treated plates that have been stained with crystal violet. (c) Approximately 10^9^ C. pseudodiphtheriticum cells were spotted on a BHIT agar plate seeded with ∼10^8^ cells of S. aureus JE2 or with strains that contain Tn insertions in *aur*, *icaR*, *splB*, *scpA*, or *rsp*. Images of the ZOC were taken after 120 hours of incubation at 28°C and are representative of three independent biological replicates. The ZOC distance was measured using ImageJ software. Each symbol represents the arithmetic mean from three independent biological replicates, and errors bars represent ±1 standard deviation. Download FIG S4, TIF file, 2.7 MB. Copyright © 2019 Hardy et al. 2019 Hardy et al. This content is distributed under the terms of the Creative Commons Attribution 4.0 International license.

### Coculture with C. pseudodiphtheriticum is detrimental to S. aureus cell surface morphology.

**FIG 8:**
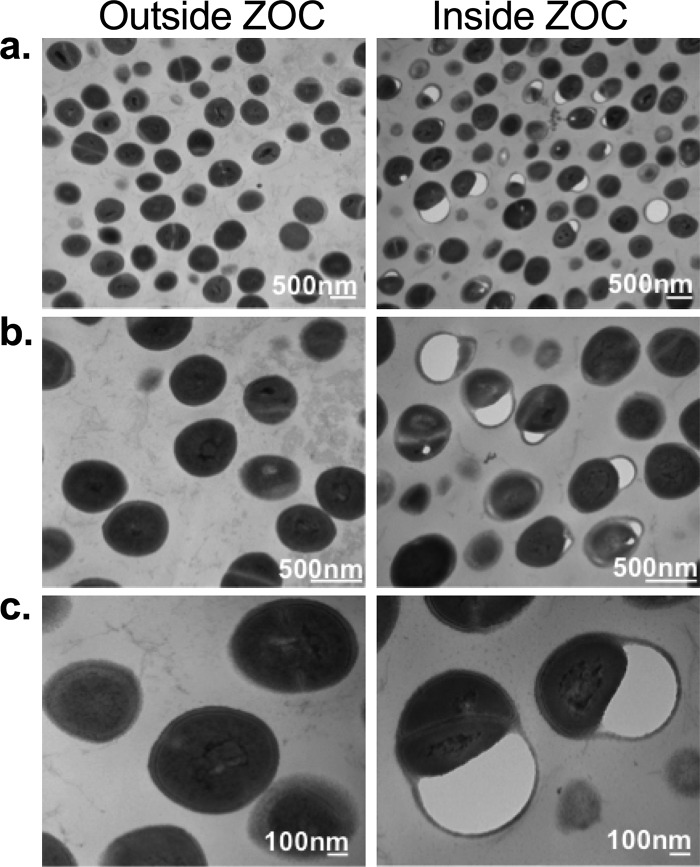
Coculture with C. pseudodiphtheriticum is detrimental to S. aureus cell morphology and integrity. S. aureus 2014.N was spread in a lawn on top of agar plates, and C. pseudodiphtheriticum was spotted directly on top. Plates were incubated at 28°C for 24 h, and cells were recovered directly adjacent to C. pseudodiphtheriticum (Inside ZOC) and elsewhere on the plate (Outside ZOC). Changes in S. aureus cell morphology were examined by transmission electron microscopy (TEM) at ×25,000 (a), ×50,000 (b), and ×100,000 (c) magnification.

Our initial bacterial interactions experiments (Fig. 1) revealed that after 48 to 72 h, a ZOC developed around the C. pseudodiphtheriticum spot, and few surviving S. aureus cells were recoverable from the ZOC (Fig. 1c). In order to directly visualize C. pseudodiphtheriticum-mediated effects on S. aureus, we slightly modified the bacterial interaction assay by swabbing S. aureus 2014.N (highly sensitive) directly on top of the agar plate and then spotting C. pseudodiphtheriticum directly on top of the S. aureus lawn. After 24 h of incubation, S. aureus cells were recovered from inside and outside the ZOC and were visualized with transmission electron microscopy (TEM). TEM analysis revealed that even after only 24 h of incubation, there were dramatic morphological changes in the S. aureus cells that were recovered from inside the ZOC compared to the cells recovered from outside the ZOC (Fig. 8a). A closer view of the cells recovered from inside the ZOC revealed that the S. aureus cell surface integrity was severely compromised and that there was some possible leakage of intracellular constituents into the extracellular space (Fig. 8b and c), possibly by plasmolysis. Thus, as suggested by the other interaction assays, C. pseudodiphtheriticum*-*mediated killing of S. aureus appeared to be due to significant damage to the S. aureus cell surface that likely leads to eventual cell lysis.

Finally, to determine whether expression of *agrBDCA*, *psm*, and *pmt* changed in response to the presence of C. pseudodiphtheriticum, qRT-PCR analysis was performed on RNA from S. aureus cells recovered inside and outside the ZOC after 3 or 24 h of coincubation: highly sensitive (2014.N), intermediately sensitive (LAC), and resistant (Mu50) strains were tested. No significant differences in expression of any of the genes at either time point were observed for 2014.N or Mu50 (Fig. S5). Conversely, S. aureus LAC cells recovered inside the ZOC showed higher levels of expression of each gene at 3 h; this was statistically significant for *psm* expression (*P* = 0.0052). This difference in transcript level was lost by 24 h, when killing begins to be readily discernible. These results suggest that though coincubation may alter gene expression in some S. aureus strain backgrounds, the overall sensitivity to C. pseudodiphtheriticum-mediated killing is not dependent on modulation of expression of any of the tested genes. Instead, the degree of killing is most closely correlated with the overall level of *agr* and *psm* expression seen in the S. aureus strain (Fig. 4 and 7).

10.1128/mBio.02491-18.6 FIG S5 Staphylococcus aureus *agr*, *psm*, and *pmt* expression upon coculture with C. pseudodiphtheriticum. Approximately 10^9^ CFU of C. pseudodiphtheriticum was spotted on a BHIT agar plate that was spread with S. aureus strains 2014.N, LAC, or Mu50. S. aureus was recovered directly next to C. pseudodiphtheriticum (In) and elsewhere on the agar plate (Out) after 3 or 24 hours of incubation at 28°C. *agrBDCA*, α-*psm*, and *pmt* gene expression levels are shown relative to 16S rRNA gene expression for three independent biological replicates. The data were plotted as follows: the geometric mean is represented as a bar, and error bars represent ±1 geometric standard deviation. A two-way ANOVA with Dunnett’s corrections for multiple comparisons was performed. The symbol above the graph ** (*P* < 0.01) indicates a statistically significant difference of α-*psm* gene expression (In versus Out) for LAC cells recovered at the 3-hour time point. Download FIG S5, TIF file, 1.0 MB. Copyright © 2019 Hardy et al. 2019 Hardy et al. This content is distributed under the terms of the Creative Commons Attribution 4.0 International license.

## DISCUSSION

Bacteria rarely exist as single species in the environments in which they live. This fact has become increasingly appreciated with the emergence of high-throughput sequencing and advances in the study of the human microbiota. Ultimately, efforts to study how bacteria interact within these polymicrobial, and often nutrient-scarce, settings have been challenging due to the fact that many bacterial species are unculturable. Furthermore, when thinking about colonization or infection of human niches, the complexity is further compounded by host factors that influence these interactions. The opportunistic pathogen S. aureus is able to successfully persist as commensal flora within the nose of some individuals; this is accomplished by mitigating the host immune response and by outcompeting other resident flora within the nasal niche (18). At any given time, one-third of the population is asymptomatically colonized with S. aureus (11, 12). However, a subset of these individuals will ultimately suffer from S. aureus-mediated disease that is often caused by the original colonizing strain (14). Therefore, it is imperative to identify the specific molecular factors that foster S. aureus commensalism versus pathogenesis. In addition, our current understanding of the complex molecular interactions that occur between the nasal microbiota and S. aureus remains limited. Thus, more detailed molecular analyses of the interspecies interactions that have been observed during microbiota studies are warranted.

Molecular profiling studies have revealed that species-specific interactions play critical roles in blocking S. aureus nasal colonization and persistence. The *Corynebacterium* genus in particular appears to greatly influence nasal S. aureus carrier status (2, 31). Indeed, both S. aureus and *Corynebacterium* species colonize the human nose (33), and there is increasing evidence that these microorganisms directly interact. Moreover, inoculation of a collection of *Corynebacterium* species into the nasal cavity was sufficient to completely eradicate S. aureus from this niche (49). Furthermore, evidence suggests that a single *Corynebacterium* species, C. pseudodiphtheriticum, is sufficient to negatively impact S. aureus nasal colonization (31). Indeed, artificial inoculation of C. pseudodiphtheriticum was sufficient to eradicate S. aureus from the nasal cavity of humans (50). However, the mechanism by which C. pseudodiphtheriticum blocks S. aureus nasal colonization remains unclear. Here, we set out to characterize the *in vitro* interaction that occurs between S. aureus and C. pseudodiphtheriticum and have sought to understand the negative interactions that occur between these species. By taking a reductionist approach, we found that C. pseudodiphtheriticum mediates specific bactericidal activity against S. aureus by ultimately affecting S. aureus cell surface integrity. Furthermore, Agr QS system-dependent regulation of the PSMs, which are important virulence factors of S. aureus, appears crucial for this activity.

Examination of the sensitivity of various S. aureus strains to C. pseudodiphtheriticum-mediated bactericidal activity revealed that the majority of tested strains were susceptible and were likely being killed by a secreted factor(s) (Fig. 1 and 2). Interestingly, there were three distinct levels of S. aureus susceptibility (Fig. 1b), which could suggest that the target(s) or mediators of bactericidal activity may be differentially expressed or functionally different between the resistant and sensitive strains. Moreover, we found that C. pseudodiphtheriticum bactericidal activity was specific to S. aureus (Fig. 1 and data not shown). This finding supports the growing body of literature that indicates that the interactions between normal flora and opportunistic pathogens are highly species specific (32, 48, 51). For example, while C. pseudodiphtheriticum mitigates S. aureus nasal colonization, the closely related *Corynebacterium* species C. accolens is associated with a higher abundance of S. aureus in the nasal cavity and appears to promote S. aureus growth *in vitro* (31). Intriguingly, C. accolens secretes a lipase that converts triacylglycerols that decorate nasal epithelial cells into free fatty acids (FFAs) and glycerol. FFAs, specifically oleic acid, have potent bactericidal activity against Streptococcus pneumoniae (51). This finding is significant because an inverse relationship exists between the presence of S. pneumoniae and S. aureus in the nasal cavity (52). Thus, some *Corynebacterium* species may influence S. aureus nasal carrier status by reducing or promoting competition from other incoming opportunistic pathogens.

Our initial characterization experiments and transposon mutant strain screen revealed that S. aureus strains that have little to no Agr QS activity, either due to insertional inactivation of *agrBDCA* via a transposition event or due to other spontaneous changes that reduced *agrBDCA* expression, are resistant to bactericidal activity (Fig. 3 and 4; see also Fig. S1 in the supplemental material). S. aureus strains N315 and Mu50, which both intrinsically express lower *agrBDCA* and RNAIII transcript levels (53), were also resistant to killing (Fig. 1 and 4). It is worth noting that during infection, Agr QS-defective strains are generally less virulent and show decreased expression of many secreted virulence factors; conversely, these strains show increased expression of many colonization factors (23, 54). The fact that only the phenotypically less virulent S. aureus strains can escape C. pseudodiphtheriticum-mediated killing perhaps suggests an interesting interplay between bacterial competition, virulence, and commensalism. In this vein, it is worth noting that the ability of *Corynebacterium* species to influence expression of S. aureus virulence factors is supported by recent work from Ramsey et al. that showed that a coculture of S. aureus and Corynebacterium striatum, another common nasal commensal species, led to global changes in S. aureus gene transcription (55). These expression changes included significant decreases in transcript levels of *agrBDCA*, *psm*β_1_, and *psm*β_2_. In contrast, transcription of genes that encode proteins that are important for colonization of nasal epithelial cells, including *Staphylococcus* protein A (*spa*) and iron-regulated surface determinant adhesin (*isdA*), was greatly increased; these expression patterns mimic S. aureus gene expression seen in the nasal cavity of cotton rats and humans (28, 29). Furthermore, spent medium derived from C. pseudodiphtheriticum led to a similar reduction in *agrBDCA* expression, indicating that S. aureus modulates gene expression in response to factors remaining in the medium and perhaps suggesting that this is a response to the presence of the *Corynebacterium* genus (55). Given the data presented here, perhaps S. aureus turns off virulence factor expression as a means to escape C. pseudodiphtheriticum-mediated killing, and thus, an inadvertent consequence of the competition between these two species is selection for S. aureus to remain in a phenotypic nonpathogenic state.

We observed that the PSMs are likely the downstream effectors of the Agr QS that are responsible for C. pseudodiphtheriticum-mediated bactericidal activity (Fig. 5, 6, and 7); S. aureus LAC strains that contained markerless deletions of all 6 *psm* genes were highly resistant to killing activity. In addition, restoration of the α*-psm* genes to resistant isolates was sufficient to restore sensitivity to C. pseudodiphtheriticum-mediated bactericidal activity, even in a different species, S. epidermidis (Fig. 6). Thus, C. pseudodiphtheriticum may target the activity of the PSMs or their transporter, PMT, as a mechanism to induce S. aureus. While the exact role of the PSMs in C. pseudodiphtheriticum-mediated bactericidal activity remains unclear, three plausible mechanisms can be gleaned from the existing literature. First, induction of aberrant accumulation of PSMs within S. aureus cells due to decreased expression of *pmt* has been shown to result in rapid killing of S. aureus. Indeed, this killing is so robust that PMT appears to be essential for S. aureus survival in the presence of the *psm* genes; *pmt* can be deleted only concurrently with deletion of all of the *psm* genes (39). Thus, perhaps C. pseudodiphtheriticum causes cell death via induction of an aberrant accumulation of the PSMs within the cell. Second, recent work by Pader et al. (56) found that strains that lack Agr QS activity show enhanced resistance to daptomycin. In Agr QS-defective strains, phospholipid shedding from the cell membrane is increased (56). These phospholipids bind to and inactivate antibiotics like daptomycin, increasing resistance. Mechanistically, this process is directly linked to the lack of PSM synthesis and secretion. Once secreted, the PSMs normally act like detergents that can break up phospholipids. Thus, if the PSMs are not synthesized, more phospholipids are shed and active, which in turn results in increased resistance to daptomycin. Thus, perhaps S. aureus increased phospholipid shedding is responsible for the resistance of the Δ*psm* mutant strain; C. pseudodiphtheriticum is producing a compound that can be bound and inactivated by shed S. aureus phospholipids. Finally, it is possible that the absence of *psm* expression indirectly stabilizes the S. aureus cell surface structure through some unknown mechanism, which results in resistance to C. pseudodiphtheriticum-mediated bactericidal activity. Future studies will seek to understand and mechanistically define the exact role of the PSMs in this process.

It is tantalizing to explore the idea that products from commensal bacteria, or the use of commensal bacteria as next-generation probiotics, could be used as novel strategies to block opportunistic pathogens. For example, Kanmani et al. found that nasal priming of C. pseudodiphtheriticum in mice led to improved resistance to respiratory syncytial virus (RSV) and subsequent Streptococcus pneumoniae superinfection (57). The proposed mechanism of resistance was via modulation of the host’s immune system, including the influx of lymphocytes. Since C. pseudodiphtheriticum can directly kill S. aureus and can modulate virulence gene expression (55), our work adds to the growing body of literature that suggests antipathogen properties of this species. Moreover, we observed that S. aureus spontaneous resistance to C. pseudodiphtheriticum-mediated bactericidal activity occurred at a relatively low frequency (~10^−8^); however, this resistance came with a cost of attenuated virulence gene expression. Deficiencies in *agrBDCA* expression result in virulence attenuation and have been shown to result in less severe S. aureus-mediated disease (23). The possible use of compounds that inhibit or decrease virulence of S. aureus as a strategy to reduce severity of disease is a relatively unexplored field. However, some work suggests that limiting quorum sensing could be one possible mechanism to decrease S. aureus virulence during active infection. Indeed, the host may seek to reduce virulence using this mechanism; human serum has been found to reduce S. aureus Agr QS activity via the sequestration of AIP (58). Recently, Paharik et al. found that a commensal *Staphylococcus* species, S. caprae, inhibits S. aureus Agr QS via the production of an inhibitory AIP (59). This reduction of S. aureus Agr QS activity resulted in reduced skin burden and necrosis. Thus, the possibility of targeting a virulence regulon as a means of controlling disease is an attractive approach. Nevertheless, there are several caveats that must be considered with this type of approach. For example, the use of inhibitory *agr* compounds as a method to block virulence does not lead to clearance of S. aureus (59). Indeed, it is well established that loss of *agr* expression promotes S. aureus biofilm formation and expression of colonization factors (29, 60); this could be particularly problematic for immunocompromised individuals since the local burden of S. aureus could be significantly increased. Alternatively, given that C. pseudodiphtheriticum directly kills S. aureus and that S. aureus seeks to escape this killing by turning off virulence gene expression, the use of C. pseudodiphtheriticum-derived products as a way to block nasal colonization and/or treat active disease may be superior to antivirulence compounds alone.

While our work sought to elucidate the molecular mechanism that C. pseudodiphtheriticum may use to prevent S. aureus nasal colonization, it is critical to note that the nasal microenvironment is complex, and bacterial species that negatively affect S. aureus nasal colonization are not limited to *Corynebacterium*. There are numerous examples of coagulase-negative *Staphylococcus* (CoNS) species that have evolved strategies to compete with S. aureus in the nose. For example, some S. epidermidis strains synthesize and secrete a serine protease, Esp, which is capable of disrupting S. aureus biofilm formation and is able to block nasal colonization (61). Staphylococcus hominis, another common commensal bacterial species, and S. epidermidis both secrete strain-specific antimicrobial peptides (AMPs) that have potent selective bactericidal activity against S. aureus (62). Furthermore, these novel AMPs were found to work synergistically with the human AMP LL-37 to prevent atopic dermatitis. Staphylococcus lugdunensis produces a novel cyclic peptide antibiotic, lugdunin, that has bactericidal properties against several Gram-positive pathogens, including S. aureus; lugdunin can prevent S. aureus nasal colonization (63). Growing evidence indicates that within the human nose there is a clear selective pressure, even among closely related commensal species, to block or eliminate S. aureus. Furthermore, compounds from commensal CoNS have been shown to eliminate S. aureus in the nasal cavity in humans (63).

There are limitations to our study. For example, while the data indicate that the PSMs are involved in C. pseudodiphtheriticum-mediated killing of S. aureus, the exact molecular mechanism by which this occurs remains to be elucidated. Furthermore, it is well recognized that *in vitro* phenotypes do not always correlate with *in vivo* phenotypes. Therefore, even though human studies suggest negative interactions between *Corynebacterium* and S. aureus (31, 34, 49, 50, 55), the observed roles of Agr QS and the PSMs in C. pseudodiphtheriticum*-*mediated killing may not be as relevant *in vivo*. Thus, the significance of our findings remains to be tested in the context of nasal colonization in an appropriate animal model. Even then, given that multispecies bacterial communities are complex and dynamic, it will be difficult to confirm that eradication of S. aureus in the nasal cavity is due solely to a C. pseudodiphtheriticum-derived factor(s); it is possible that a C. pseudodiphtheriticum-derived factor(s) may synergize with other host or bacterium-derived compounds to ultimately lead to S. aureus killing. Clearly, identification and purification of the C. pseudodiphtheriticum-produced bactericidal factor(s) will aid investigation of these possibilities.

In summary, our study has revealed that the molecular interactions between *Staphylococcus* and *Corynebacterium* are far more multifaceted than previously recognized. From the results presented here, we conclude that C. pseudodiphtheriticum selectively targets S. aureus for killing. This works expands on the well-established negative correlation between the presence of C. pseudodiphtheriticum and S. aureus in the nose that has been observed in numerous nasal microbiota cohort studies (31, 50). Moreover, given our finding that strains that were deficient in Agr QS were resistant to C. pseudodiphtheriticum-mediated killing, there may be a selective advantage for S. aureus strains to switch from a pathogenic state to a commensal state. Thus, in order to coexist with C. pseudodiphtheriticum and to increase the probability of successful colonization of the nasal niche, S. aureus must surrender expression of its primary virulence factors. Future work will pursue the identity of the C. pseudodiphtheriticum-derived factor(s) that facilitates bactericidal activity against S. aureus. *En masse*, our findings support the continued study of “bacterial warfare” between commensal and pathogenic bacterial species as a way to develop novel therapeutics and/or identify molecular vulnerabilities of pathogens.

## MATERIALS AND METHODS

### Strains, culture, and bacterial interaction assays.

Strains used in this study are listed in Table 1, and primers and plasmids are listed in Table 3. All S. aureus strains were typed for the *agr* allele using established protocols (38). Corynebacterium pseudodiphtheriticum strain USU1 was isolated as part of a cooperative agreement between the Uniformed Services University of the Health Sciences and the Walter Reed Military Medical Center Clinical Microbiology Lab. Strains were maintained as −80°C freezer stocks and were revived as follows: *Corynebacterium* species were streaked from frozen glycerol stocks on brain heart infusion (BHI) agar (Becton, Dickinson) with 1% Tween 80 (BHIT; Sigma-Aldrich), and *Staphylococcus* species were streaked from glycerol stocks on BHI agar. Each strain was expanded by overnight incubation at 35°C. The bacterial interaction assay was adapted from published protocols (31). Briefly, 0.04 g of S. aureus or S. epidermidis was directly harvested from agar plates with a sterile inoculating loop and then resuspended in 0.2 ml of sterile 0.9% NaCl (Fisher Chemicals). Eight microliters (∼10^8^ CFU) of the cell suspension was then inoculated into 15 ml of autoclaved BHIT agar that had been precooled to 55°C; inoculated agar was then poured into a sterile petri dish. The agar was allowed to cool in a laminar flow hood for 30 min. Next, 25 μl of a C. pseudodiphtheriticum, C. accolens, or C. diphtheriae (∼5 × 10^9^ CFU) cell suspension was spotted onto the center of the agar and was allowed to dry in the laminar flow hood for 40 min. The resulting plates were incubated at 28°C, and the zone of clearance (ZOC) was examined every 24 h for a total of 120 h. The ZOC was defined as the distance between the edge of the *Corynebacterium* spot and the visible edge of the clearance ring. To measure the ZOC, images were analyzed using ImageJ software (64). Unless otherwise noted, a two-way analysis of variance (ANOVA) statistical test with Dunnett’s correction for multiple comparisons was used to assess significance; a confidence interval of 95% and an alpha value of 0.05 were considered significant.

**TABLE 3:** Primers and plasmids used in this study

Oligonucleotide or plasmid	Sequence (5′–3′) or notes	Reference
Oligonucleotides		
Quantitative real-time PCR		
*agrB* FWD	GACCAGTTTGCCACGTATCT	This study
*agrB* REV	GCTAAGACCTGCATCCCTAATC	This study
RNAIII FWD	GGAGTGATTTCAATGGCACAAG	This study
RNAIII REV	GTGAATTTGTTCACTGTGTCGATAA	This study
*psm*α_1_ FWD	GGTATCATCGCTGGCATCATT	This study
*psm*α_1_ REV	CCATGTGAAAGACCTCCTTTGT	This study
*pmtA* FWD	CTTGCGCATGTTCTGTTAATCC	This study
*pmtA* REV	TCAAGTTGTGAGTGGTGCTATT	This study
16S rRNA FWD	GTGGAGGGTCATTGGAAACT	This study
16S rRNA REV	CACTGGTGTTCCTCCATATCTC	This study
*agrC* complementation		
*agrC* FWD BamHI	TGGATCCAGAGGAGAAATTAAATGAT	This study
*agrC* REV SstI	CCCGAGCTCCTAGTTGTTAATAATTTCAAC	This study
Transposon insertion sequencing		
RP45	GAGTGTGATGATAAGTGGGAAGGAC	This study
SS1905	GGTAAACTATGATTCACGACGACTAG	This study
Plasmids		
pCL15	E. coli-S. aureus shuttle vector	70
pCL15-P_spac_-*agrC*	*agrC* complementation vector	This study
pTX_Δ_*psm*α1-4	*psm* complementation vector	39
pTX_Δ_16	Empty vector	81

To determine whether live C. pseudodiphtheriticum was required to mediate bactericidal activity, ∼5 × 10^9^ CFU of C. pseudodiphtheriticum was heated to 90°C for 10 min; loss of viability was confirmed by plating on BHIT agar. The heat-killed C. pseudodiphtheriticum was inoculated onto S. aureus-seeded plates as described above. Images were taken after 24 and 120 h of incubation at 28°C. To determine if bactericidal activity was dependent on direct physical contact, a sterile 0.2-μm filter disk was placed on top of the BHIT agar that had been seeded with S. aureus; C. pseudodiphtheriticum was then spotted on top of the filter disk. Images of the ZOC were taken after 24 and 48 h of incubation at 28°C.

### *Corynebacterium* CCFM preparation and disk diffusion assay.

Cell-free conditioned medium (CCFM) was prepared from C. pseudodiphtheriticum according to the method of Ramsey et al. (55) with several modifications. One-liter C. pseudodiphtheriticum cultures were grown for 24 h in BHIT broth at 37°C with shaking at 190 rpm. Cultures were centrifuged at 13,000 rpm for 10 min, and culture supernatant was then passed through a 0.2-μm filter (Corning). Sterile CFCM was further concentrated by ammonium sulfate precipitation. Sterile saturated ammonium sulfate was added to sterile CFCM at 1× the original volume. The suspension was centrifuged at 13,000 rpm for 10 min, and the supernatant was decanted. The pellet was air dried and resuspended in 1× PBS (Fisher Chemicals) at a 50× final concentration. For the disk diffusion assay, S. aureus was cultured in BHI broth overnight at 37°C. The following day, these cultures were diluted to 1 × 10^8^ cells/ml (OD_600_ of 0.1) in BHI broth, and a sterile swab was used to spread the S. aureus cell suspension on BHIT agar as a lawn. The plate was allowed to dry in a laminar flow hood for 30 min. Next, a sterile 6-mm diffusion disk was place on top of the S. aureus lawn, and 50 μl of CCFM was inoculated onto the disk. Plates were incubated at 28°C, and images were taken after 24 and 72 h of incubation.

### Recovery of resistant S. aureus isolates.

S. aureus isolates that developed spontaneous resistance to C. pseudodiphtheriticum-mediated killing were recovered by extracting 5-mg punches of agar from the ZOC directly adjacent to the C. pseudodiphtheriticum spot with a sterile pipette tip. To estimate the frequency of resistance, comparable punches of agar were also taken from an area directly outside the ZOC. Agar punches were resuspended in 1 ml of BHI and heated to 55°C for 10 min. The entire 1-ml cell suspension was plated on mannitol salt agar (MSA; Criterion). Plates were incubated at 37°C overnight, and recovered colonies were visualized. The frequency of spontaneous resistance to bactericidal activity was calculated by dividing the number of S. aureus CFU recovered from a 5-mg punch inside the ZOC by the number of S. aureus CFU recovered from a 5-mg punch directly outside the ZOC. To confirm that S. aureus colonies recovered from the ZOC were stably resistant to C. pseudodiphtheriticum-mediated killing, five recovered colonies were chosen at random and reassayed in the described bacterial interaction assay.

### Screen for resistant S. aureus transposon mutant strains.

Transposon vector pRP1313 was engineered by adding features of the *mariner* C9 transposase from pMarB (65) to pMAD (66) as described in Text S1 and Table S1 in the supplemental material. The vector was transformed into RN4220 (yielding strain SAP370) and transduced into NRS384 (yielding strain SAP372) as previously described (67, 68), using chloramphenicol (10 µg/ml) as the selectable marker and incubating cultures at 30°C.

10.1128/mBio.02491-18.1 TEXT S1 Supplemental materials and methods. Download Text S1, DOCX file, 0.03 MB. Copyright © 2019 Hardy et al. 2019 Hardy et al. This content is distributed under the terms of the Creative Commons Attribution 4.0 International license.

10.1128/mBio.02491-18.7 TABLE S1 Oligonucleotides used in the construction of the transposon vector. Download Table S1, DOCX file, 0.02 MB. Copyright © 2019 Hardy et al. 2019 Hardy et al. This content is distributed under the terms of the Creative Commons Attribution 4.0 International license.

To generate a pooled S. aureus transposon library, a 5-ml culture of SAP372 was grown overnight at 30°C in tryptic soy broth (TSB) containing chloramphenicol (10 µg/ml). The overnight culture was subcultured at 1:1,000 in TSB without antibiotics and was incubated for 1 h at 43°C. Spectinomycin was added (250 µg/ml), and the culture was incubated for an additional 6 h at 43°C. A spiral plater (Spiral Biotech AP-5000 Autoplate system) was used to spread 20 µl of this culture onto each of 48 tryptic soy agar plates containing spectinomycin (250 µg/ml), which were incubated overnight at 43°C. Growth on the plates was pooled (eight pools of six plates each) and resuspended in 30 ml of TSB per pool (∼6,000 colonies per pool). Suspensions were centrifuged for 13 min at 4,700 rpm. Supernatants were poured off, and each pellet was resuspended in 32.5 ml of TSB containing 15% glycerol. Aliquots of 1 ml were stored at −80°C.

To identify transposon mutant strains that were resistant to C. pseudodiphtheriticum-mediated bactericidal activity, the pooled library was used to seed the agar as described for the bacterial interaction assay and surviving transposon mutant strains were recovered from within the ZOC as described above. Each recovered transposon mutant strain was independently confirmed to be stably resistant to C. pseudodiphtheriticum-mediated bactericidal activity. To identify the region of transposon insertion, a rescue cloning strategy was utilized that relied upon the presence of an R6K origin of replication in the *H1 mariner* transposon. Briefly, each resistant transposon mutant strain was grown overnight in 2 ml of BHI broth containing 50 µg/ml of spectinomycin (Sigma-Aldrich) at 37°C with shaking. Cells were pelleted by centrifugation and resuspended in 0.2 ml of 1× phosphate-buffered saline (PBS; Gibco) with 10 μg/ml lysostaphin (Sigma-Aldrich). The cell suspension was incubated at 37°C for 1 h. DNA was then extracted using the Wizard Genomic DNA purification kit (Promega) according to the manufacturer’s instructions. Recovered DNA was digested with MfeI-HF (New England Biolabs), and a self-ligation was performed via the addition of Quick ligase (New England Biolabs). The resulting material was then transformed into chemically competent DH5α λpir, and colonies containing plasmids were recovered by plating on BHI agar that contained 50 µg/ml spectinomycin. Spectinomycin-resistant colonies were subcultured overnight in BHI broth containing 50 µg/ml spectinomycin. Plasmid DNA was then extracted from these cultures using the QIAprep Spin miniprep kit according to the manufacturer’s instructions (Qiagen). The site of transposon insertion was determined by sequencing each plasmid in the forward (primer RP45) and reverse (primer SS1905) directions. The resulting sequences were then aligned against the S. aureus NCTC 8325 reference genome deposited in GenBank (69).

### *agrC* complementation.

Purified JE2 genomic DNA was used to amplify *agrC* using primers *agrC* FWD BamHI and *agrC* REV SstI with the following conditions: an initial 30 s denaturation step at 98°C, followed by 35 cycles (10 s of denaturation at 98°C, 30 s annealing at 50°C, and 45 s of extension at 72°C), and a final extension step at 72°C for 10 min. The PCR fragment was digested with BamHI and SstI and was ligated into similarly digested pCL15 (70). In the resulting plasmid, pCL15-P_spac_-*agrC*, *agrC* is under the control of the P_spac_ promoter. The resulting ligation was transformed into chemically competent Escherichia coli TOP10 cells, and colonies containing pCL15-P_spac_-*agrC* were selected for on BHI agar containing 100 µg/ml ampicillin. To confirm plasmid integrity, pCL15-P_spac_-*agrC* was purified and sequenced prior to being electroporated directly into JE2 *Tn*::*agrC.* S. aureus transformants were selected on BHI agar containing 25 µg/ml chloramphenicol. Colony PCR on chloramphenicol-resistant colonies with primers *agrC* FWD BamHI and *agrC* REV SstI was used to confirm the presence of *agrC*. The complemented strain JE2 *Tn*::*agrC*-pCL15-P_spac_-*agrC* is referred to as JE2 *Tn*::*agrC*^C^ throughout this study.

### Creation of S. aureus JE2 *Tn*::*agrC* pTX_Δ_*psm*α1-4 and S. epidermidis pTX_Δ_*psm*α1-4.

LACΔ*psm* pTX_Δ_*psm*α1-4 was cultured overnight at 37°C in BHI broth containing 10 µg/ml tetracycline (Sigma). Cells were pelleted by centrifugation, and pTX_Δ_*psm*α1-4 was extracted using the QIAprep Spin miniprep kit according to the manufacturer’s instructions (Qiagen), with the following exceptions: 10 μg/ml lysostaphin was added to the P1 buffer, and the cell suspension was incubated at 37°C for 30 min. Purified plasmid was then transformed into electrocompetent JE2 *Tn*::*agrC* or S. epidermidis 1457 cells. Transformants were selected for on LB agar containing 10 μg/ml tetracycline. To confirm that the plasmid was stably present in transformants, plasmid was then reisolated from JE2 *Tn*::*agrC* pTX_Δ_*psm*α1-4 and S. epidermidis 1457 pTX_Δ_*psm*α1-4 cells as described above. Purified pTX_Δ_16 plasmid was transformed into electrocompetent S. epidermidis 1457 to generate S. epidermidis 1457 pTX_Δ_16, an empty vector control. Transformants were selected for on LB agar containing 10 μg/ml tetracycline.

### RNA extraction, cDNA synthesis, and qRT-PCR analysis.

Parental S. aureus strains and resistant derivative isolates were cultured on fresh BHI agar plates at 28°C overnight (16 to 18 h) or cocultured with C. pseudodiphtheriticum on BHIT agar plates for 3 or 24 h at 28°C. A sterile inoculating loop was used to resuspend cells in 1 ml of freshly prepared lysis buffer (1× TE buffer with 10 μg/ml lysostaphin and 20 μg/ml proteinase K). The cell suspension was incubated at 37°C until the cell suspension was clear (5 to 15 min). RNA was isolated using TRIzol reagent (Invitrogen) as described previously (71), with the following exceptions: the aqueous phase was used directly in an RNeasy cleanup protocol (Qiagen), and a 60-min DNase (Qiagen) on-column digestion was performed during the cleanup. cDNA was synthesized using the QuantiTect reverse transcription kit (Qiagen) according to the manufacturer’s instructions. Quantitative reverse transcription-PCR (qRT-PCR) was performed as previously described (71) and involved a Roto-Gene Q instrument (Qiagen); 1× SYBR green RT-PCR master mix (Qiagen); 3 pmol each of the forward and reverse primers (Table 2), which were designed with the IDT PrimerQuest Suite; and 1 μl of cDNA. qRT-PCR primers were confirmed by sequencing analysis to be 100% homologous to the corresponding gene across all strains tested. A reaction mixture in which no reverse transcriptase enzyme was added was included as a no-RT control. Cycling conditions were as follows: an initial activation step for 5 min at 95°C and 35 cycles of denaturation at 95°C for 10 s, annealing at 60°C for 20 s, and extension at 72°C for 10 s. SYBR green fluorescence was measured during each extension step. Transcript level, 2^−ΔCT^, is presented relative to the 16S rRNA gene. Unless otherwise noted, a two-way analysis of variance (ANOVA) statistical test with Dunnett’s correction for multiple comparisons was used to assess significance; a confidence interval of 95% and an alpha value of 0.05 were considered significant.

### α-PSM production and quantification.

S. aureus strains 2014.N and 2014.N Survivor A were cultured in BHIT broth at 28°C overnight (16 to 18 h) with gentle shaking. Cells were pelleted by centrifugation, and the culture supernatant was filter sterilized with a 0.2-μm filtration unit. Total relative α-PSM concentrations for each culture was determined as described using reverse-phase high-pressure liquid chromatography–electrospray mass spectrometry (RP-HPLC/ESI-MS) (22). Total α-PSM is reported as CEICS peak area. To determine if total α-PSM was significantly different between strains, a two-tailed Student *t* test with Welch’s correction was utilized.

### Transmission electron microscopy.

C. pseudodiphtheriticum USU1 was streaked on BHIT agar, and S. aureus 2014.N was streaked on BHI agar. Each strain was expanded by overnight incubation at 35°C. Following incubation, 0.04 g of S. aureus 2014.N was directly harvested from agar plates with a sterile inoculating loop and then resuspended in 0.2 ml of sterile 0.9% NaCl. A sterile swab was used to spread the S. aureus cell suspension as a lawn on BHI agar. The plate was allowed to dry in a laminar flow hood for 30 min. Next, 25 μl of a C. pseudodiphtheriticum cell suspension, prepared as described above, was spotted onto the center of the agar and was allowed to dry in the laminar flow hood for 40 min. The resulting plates were incubated at 28°C for 24 h to allow for initiation of the development of the ZOC. S. aureus 2014.N cells directly adjacent to the C. pseudodiphtheriticum spot (inside ZOC) and cells elsewhere on the plate (outside ZOC) were picked up with a sterile cotton swab, directly resuspended in fixing solution (2% formaldehyde freshly prepared from paraformaldehyde crystals and 2% glutaraldehyde in 0.1 M cacodylate buffer, pH 7.4), and incubated for 1 h at room temperature. Fixed cells were pelleted by centrifugation, and the supernatant was decanted. The samples were washed three times for 10 min each in cold 0.1 M cacodylate buffer. Samples were incubated in 2% OsO_4_ in 0.1 M cacodylate buffer (0.1 M, pH 7.4) for 1 h and then washed three times for 10 min each in cacodylate buffer. Samples were then dehydrated in a graduated series of ethanol in water (once for 10 min each in 30%, 50%, 70%, and 95% ethanol and twice for 10 min in 100% ethanol). Following dehydration, samples were infiltrated in a graduated series of Spurr’s epoxy resin (Electron Microscopy Sciences) and then polymerized at 70°C for 11 h. Polymerized blocks were sectioned in a Leica UC6 ultramicrotome, and thin sections were collected on 3-mm copper grids. Grids were poststained in a Leica EM AC20 and then examined on a JEOL JEM-1011 transmission electron microscope (JEOL USA). Images were collected on an Advanced Microscopy Techniques (AMT Corp.) digital camera.
