# Supplementary figures and images for: Corynebacterium pseudodiphtheriticum Exploits Staphylococcus aureus Virulence Components in a Novel Polymicrobial Defense Strategy
Source: mBio. 2019 Jan 8;10(1):e02491-18. doi: 10.1128/mBio.02491-18 (PMC6325251; doi:10.1128/mBio.02491-18)

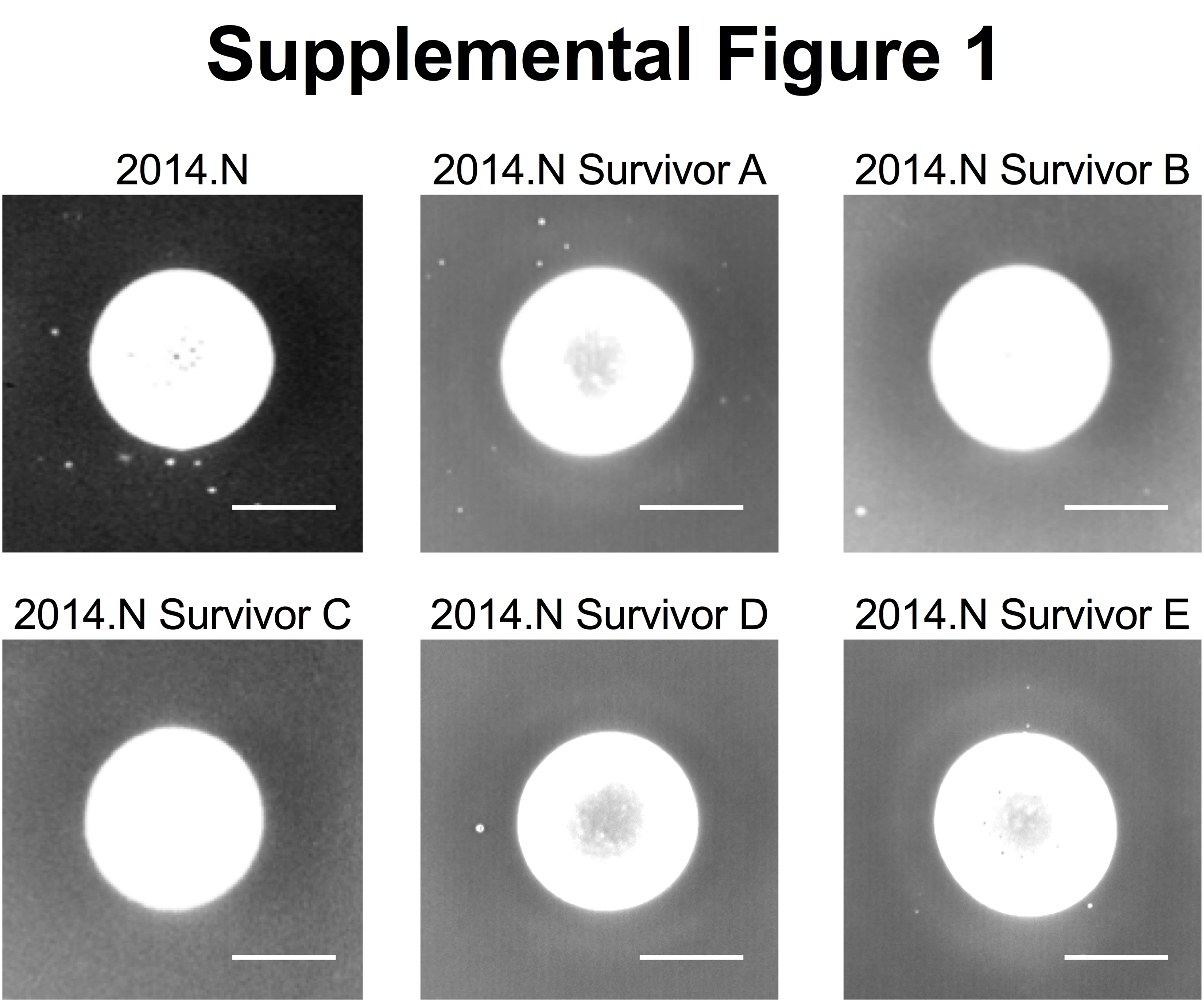

Supplement: FIG S1 [file mBio.02491-18-sf001.tif]

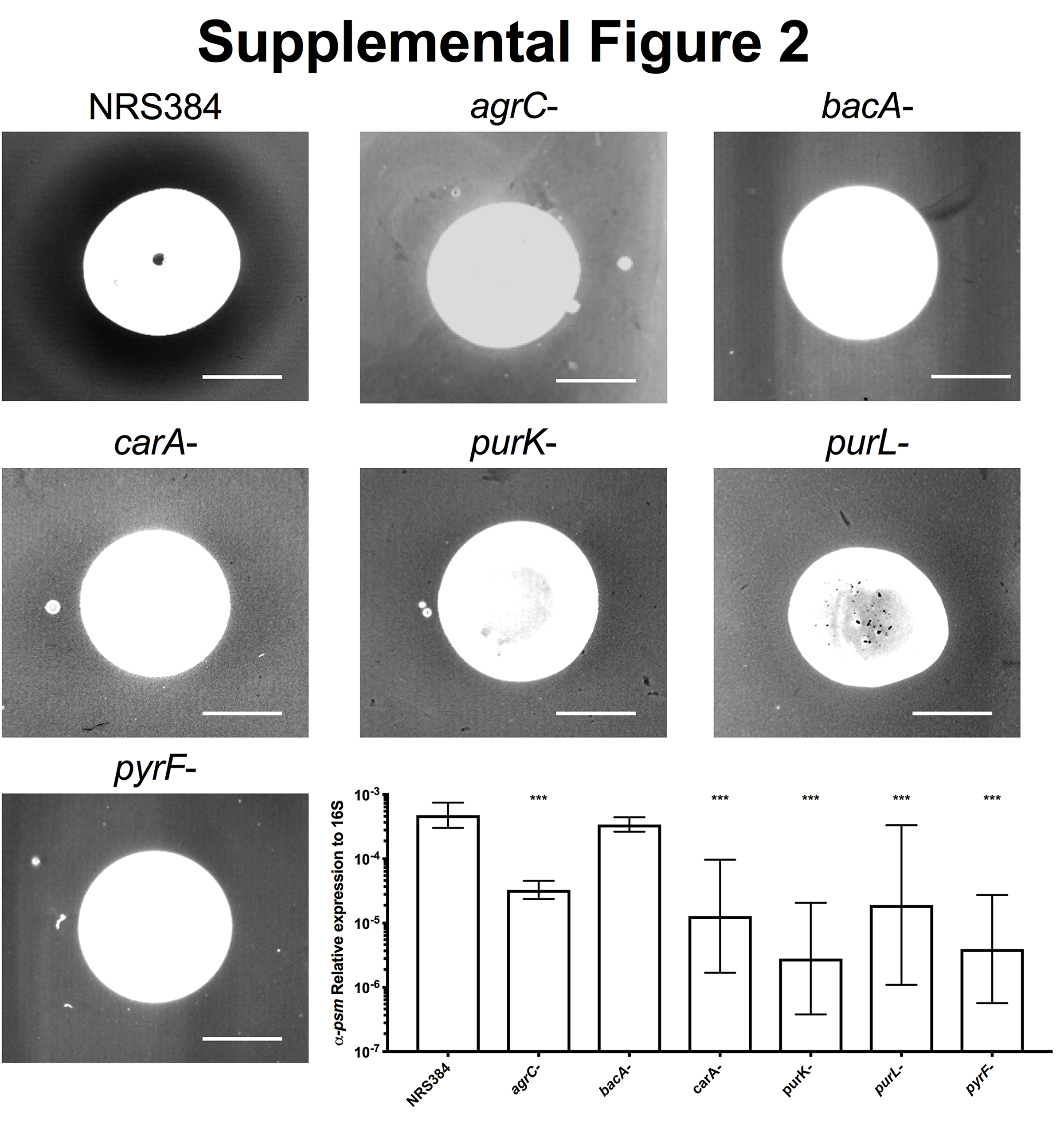

Supplement: FIG S2 [file mBio.02491-18-sf002.tif]

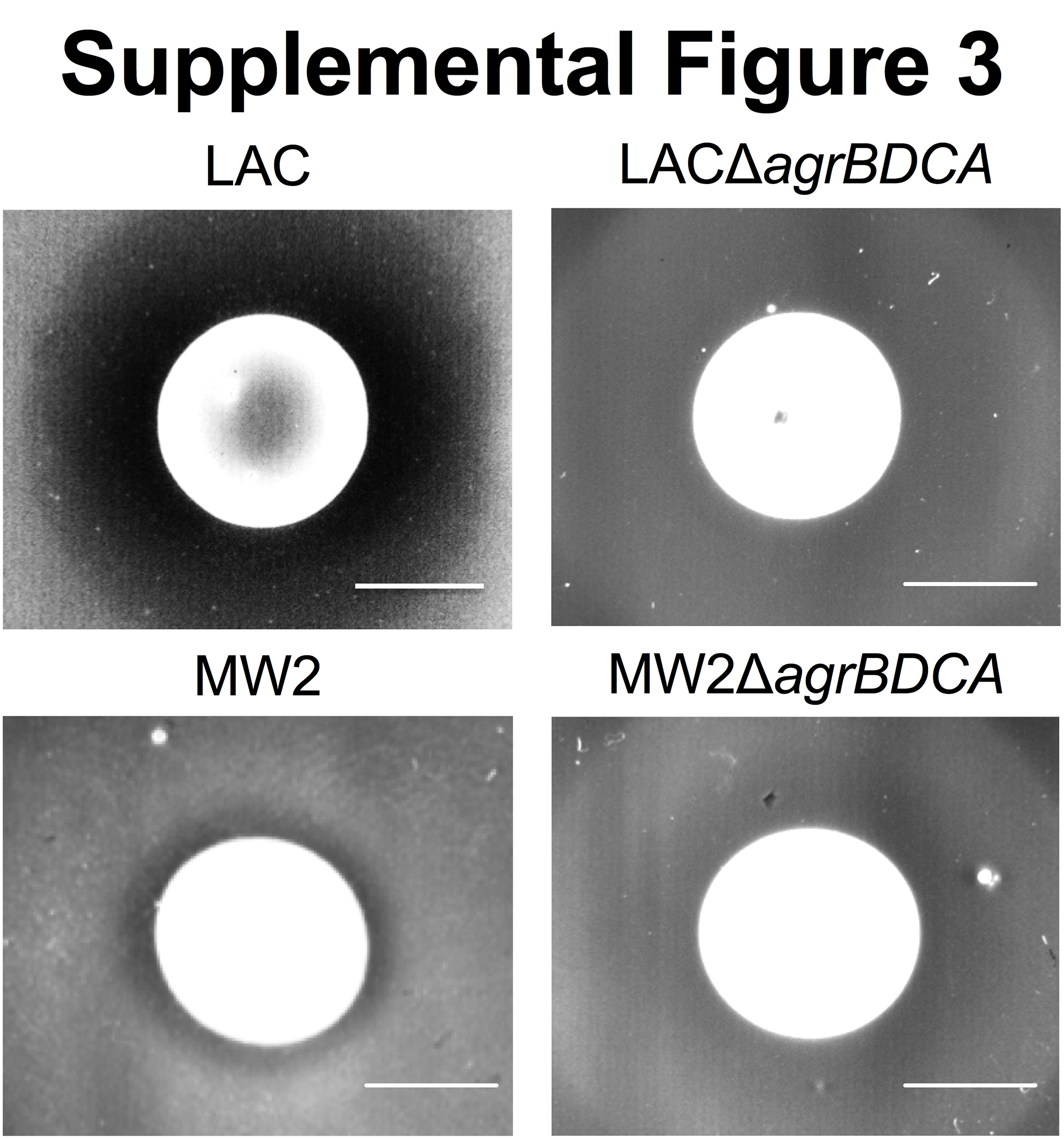

Supplement: FIG S3 [file mBio.02491-18-sf003.tif]

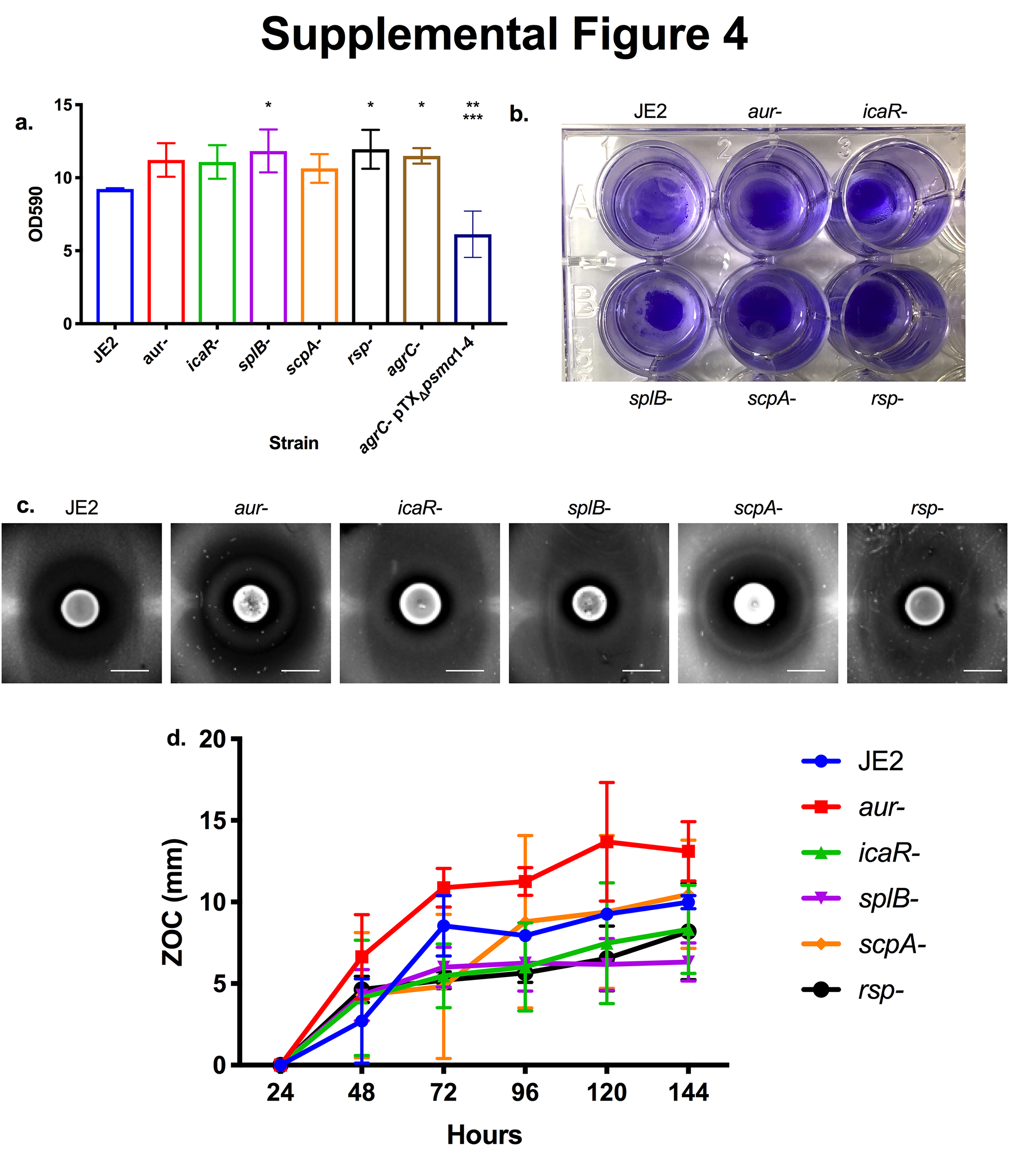

Supplement: FIG S4 [file mBio.02491-18-sf004.tif]

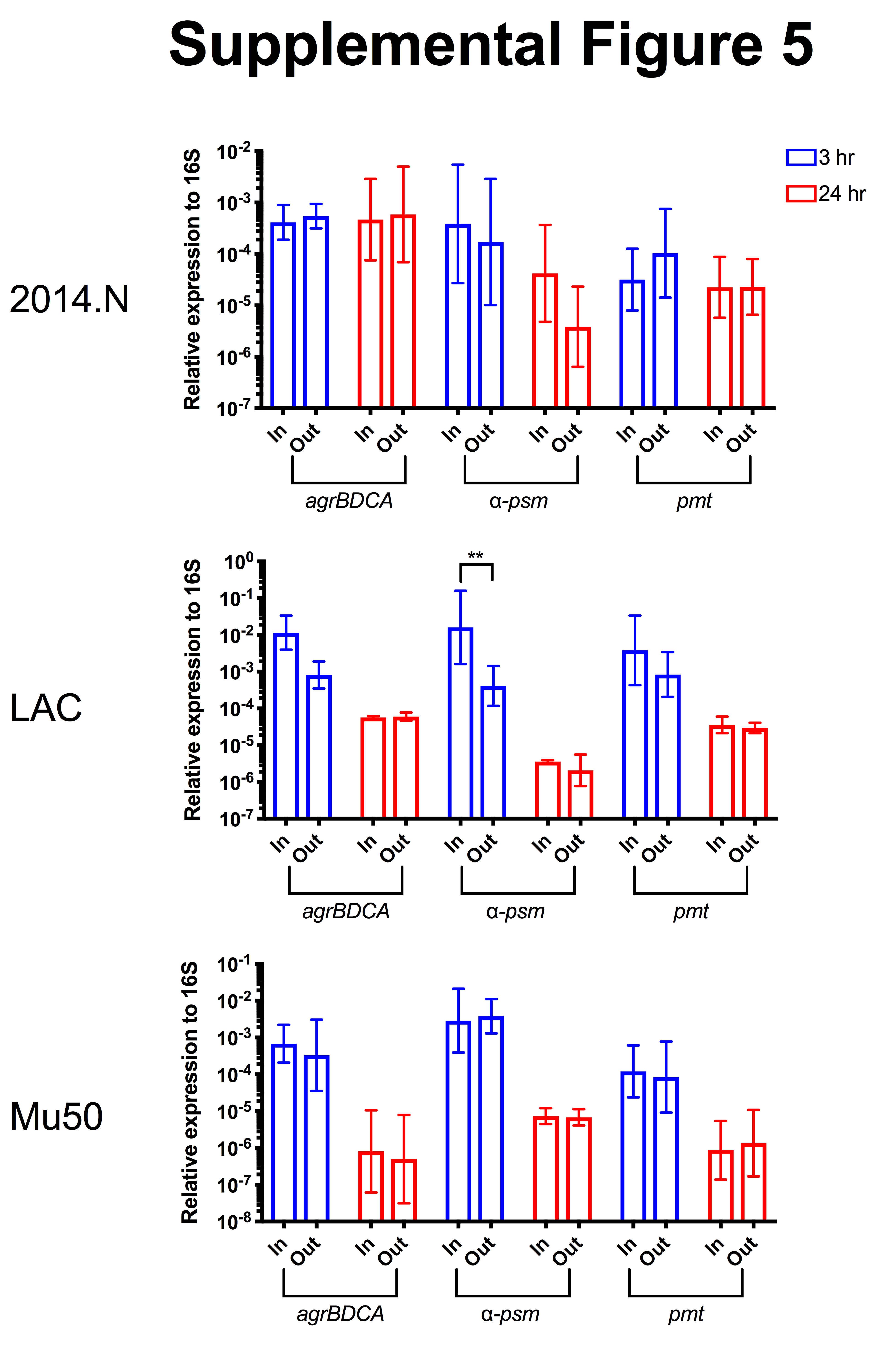

Supplement: FIG S5 [file mBio.02491-18-sf005.tif]
