# Supplementary material for: Corynebacterium pseudodiphtheriticum Exploits Staphylococcus aureus Virulence Components in a Novel Polymicrobial Defense Strategy
Source: mBio. 2019 Jan 8;10(1):e02491-18. doi: 10.1128/mBio.02491-18 (PMC6325251; doi:10.1128/mBio.02491-18)
Supplement: TEXT S1 [file mBio.02491-18-s0001.docx]

**Supplemental Materials and Methods**

**Development of transposon vector pRP1313**

All oligonucleotides used in the construction of the transposon vector are listed in Supplementary Table 1. The backbone of the transposon vector (initially developed for use in *B. anthracis*) was a plasmid pSS4030 (1), a derivative of pBKJ236 (2). To remove undesirable restriction sites, pSS4030 was digested with *PstI* and *NsiI* and self-ligated, yielding plasmid pSS4247. To add an *EagI* site, this plasmid was digested with *EcoRI* and ligated with the self-complementary oligonucleotide SS1408, yielding plasmid pSS4248. To remove *oriT* and undesirable restriction sites, pSS4248 was digested with *SalI* and *XhoI* and self-ligated, yielding pSS4249. To add back *oriT*, primers SS1454 and SS1455 were used in a PCR reaction with pBKJ236 as template. The resulting PCR product was digested with *KpnI* and was cloned into *KpnI*-digested pSS4249, resulting in plasmid pSS4341. Separately, transposon vector pMarB (3) was digested with *PstI* and ligated with the self-complementary oligonucleotide SS1404, adding an *EagI* site downstream of the C9 transposase and yielding pSS4250. To add another *EagI* site upstream of the transposase, the spectinomycin resistance gene *aad9* (originally from Tn554 (4) and its promoter region were amplified using primers SS1429 and SS1430. This PCR product was digested with *EcoRI* and was inserted into pSS4250 that had been partially digested with the same enzyme, yielding pSS4252. Digestion of this plasmid with *EagI* led to isolation of a fragment including the C9 transposase and upstream sequences; this fragment was inserted into the *NotI* site of pSS4341, yielding pSS4343. To add a marker for transposition, the kanamycin resistance gene *aphA* and its promoter region were amplified from pMarB using primers RP52 and RP53. The resulting PCR product was digested with *PstI* and inserted at the same site in pSS4343, yielding pRP1026. To add an inverted repeat, this plasmid was digested with *XbaI* and *NotI* and ligated with the complementary oligonucleotides RP58 and RP59, yielding pRP1029. To delete the *BamHI* site, the C9 transposase region was amplified using primers RP68 and RP69. The resulting PCR product was digested with *BsaI* and ligated with pRP1029 that had been digested with *BglII* and *XmaI*, yielding pRP1030. To add an additional inverted repeat, this plasmid was digested with *XbaI* and *NotI* and ligated with oligonucleotides RP58 and RP59, yielding pRP1070. To add the R6Kγ origin of replication and enhancer, these features were amplified from plasmid pJM703.1(5) using primers RP193 and RP270. The resulting PCR product was digested with *BsaI* and ligated with pRP1070 that had been digested with *AvrII* and *MluI*, yielding pRP1116, which is suitable for use in *B. anthracis.*

For a transposon vector for use in *S. aureus,* we used the backbone of pMAD (6). A 1.1-kb fragment of pRP1116, including the R6Kγ origin of replication and enhancer, one inverted repeat, and the C9 transposase, was amplified from pRP1116 using primers RP462 and RP463. This PCR product was digested with *BsaI* and ligated with pMAD that had been digested with *HindIII* and *MluI*, yielding pRP1269. To insert a selectable marker within the transposable element, the spectinomycin resistance gene *aad9* and its promoter region were amplified from pRP1028 (1) using primers RP490 and SS1400. The resulting PCR product was digested with *BsaI* and *XmaI* and ligated with pRP1269 that had been digested with *EagI* and *XmaI*, yielding pRP1280. To add a second inverted repeat, this plasmid was digested with *EagI* and *KpnI* and ligated with oligonucleotides RP491 and RP492, yielding plasmid pRP1290. To enable use of the vector in strains that are resistant to erythromycin, pRP1276 (7) was digested with *NgoMIV* and *BamHI*, and a resulting 983-bp fragment containing the chloramphenicol resistance gene *cat* and its promoter region was ligated with pRP1290 that had been digested with the same enzymes, yielding plasmid pRP1293. To enable screening of strains for the presence of the plasmid, the beta-galactosidase gene *bgaB* under the control of the constitutive *clpB* promoter (6) was amplified from pMAD using primers RP426 and RP495. The resulting PCR product was digested with BsaI and ligated with pRP1293 that had been digested with NgoMIV, yielding plasmid pRP1295. Sequencing analysis of this plasmid indicated that a 22-bp deletion in the R6Kγ origin of replication had occurred during the construction of predecessor plasmid pRP1280. To repair this deletion, a 2-kb fragment was amplified from pRP1269 using primers RP378 and RP473. This PCR product was digested with *BamHI* and *XmaI* and ligated with similarly digested pRP1295, yielding plasmid pRP1300. To add an *MmeI* site to one of the inverted repeats (for potential use for Tn-seq (8)), a 1.3-kb region containing the C9 transposon and one inverted repeat was amplified from pRP1300 using primers RP540 and RP541, digested with *BamHI* and *BsaI*, and ligated with pRP1300 that had been digested with *MluI* and *BamHI*, yielding the final transposon vector pRP1313.

**Biofilm Assay**

*S. aureus* JE2 and JE2 transposants were cultured overnight in BHIT broth at 37°C with shaking. Overnight cultures were then diluted to an OD_600_ of 0.05 in BHIT broth and were incubated at 37°C with shaking until the OD_600_ reached 1 (~1.0 X10^9^ CFU/mL). The cell suspension was then diluted to an OD_600_ of 0.1 (~1.0 X10^8^ CFU/mL) with BHIT Broth. Next, 1mL of the OD_600_ 0.1 cell suspension was transferred to a tissue culture treated 24-well plate (1 strain/well) and incubated statically at 28°C for 48 hours. Following incubation, spent medium was aspirated from each well and biofilms were gently washed three times with sterile 1X PBS. Biofilms were then allowed to dry at room temperature for 10 minutes and were subsequently fixed with 100% methanol (Sigma-Aldrich) for 5 minutes. Excess methanol was decanted and 0.5mL of crystal violet solution (Sigma-Aldrich) was added to each well, allowed to incubate for 15 minutes, and then subsequently decanted. The wells were gently washed three times with water and were allowed to dry. To solubilize the crystal violet contained within the biofilms, 0.5mL differentiation solution (Sigma-Aldrich) was added to each well for 1 minute; samples were then further diluted with 100% ethanol (Sigma-Aldrich) to obtain absorbance values within the linear range of the spectrometer. 1 mL of solubilized crystal violet solution was then transferred to a cuvette, and the absorbance (OD_590_) for each sample was read (9). A two-way ANOVA with Dunnett’s corrections for multiple comparisons was performed on OD_590_ values.

**References Cited**

1. Plaut RD, Stibitz S. 2015. Improvements to a Markerless Allelic Exchange System for *Bacillus anthracis*. PLoS One 10:e0142758.

2. Janes BK, Stibitz S. 2006. Routine Markerless Gene Replacement in *Bacillus anthracis*. Infection and Immunity 74:1949-1953.

3. Le Breton Y, Mohapatra NP, Haldenwang WG. 2006. In Vivo Random Mutagenesis of *Bacillus subtilis* by Use of TnYLB-1, a mariner-Based Transposon. Applied and Environmental Microbiology 72:327-333.

4. Murphy E. 1985. Nucleotide sequence of a spectinomycin adenyltransferase AAD(9) determinant from *Staphylococcus aureus* and its relationship to AAD(3") (9). Mol Gen Genet 200:33-9.

5. Miller VL, Mekalanos JJ. 1988. A novel suicide vector and its use in construction of insertion mutations: osmoregulation of outer membrane proteins and virulence determinants in *Vibrio cholerae* requires *toxR*. J Bacteriol 170:2575-83.

6. Arnaud M, Chastanet A, Debarbouille M. 2004. New vector for efficient allelic replacement in naturally nontransformable, low-GC-content, gram-positive bacteria. Applied and Environmental Microbiology 70:6887-6891.

7. Prabhakara R, Foreman O, De Pascalis R, Lee GM, Plaut RD, Kim SY, Stibitz S, Elkins KL, Merkel TJ. 2013. Epicutaneous model of community-acquired *Staphylococcus aureus* skin infections. Infect Immun 81:1306-15.

8. Goodman AL, McNulty NP, Zhao Y, Leip D, Mitra RD, Lozupone CA, Knight R, Gordon JI. 2009. Identifying genetic determinants needed to establish a human gut symbiont in its habitat. Cell Host Microbe 6:279-89.

9. Servetas SL, Carpenter BM, Haley KP, Gilbreath JJ, Gaddy JA, Merrell DS. 2016. Characterization of Key *Helicobacter pylori* Regulators Identifies a Role for ArsRS in Biofilm Formation. Journal of Bacteriology 198:2536-2548.
