# Supplementary material for: Corynebacterium pseudodiphtheriticum Exploits Staphylococcus aureus Virulence Components in a Novel Polymicrobial Defense Strategy
Source: mBio. 2019 Jan 8;10(1):e02491-18. doi: 10.1128/mBio.02491-18 (PMC6325251; doi:10.1128/mBio.02491-18)
Supplement: TABLE S1 [file mBio.02491-18-st001.docx]

**Supplemental Table 1: Oligonucleotides used in the construction of the transposon vector**

| Name | Sequence | Use | Restriction site(s) |
| --- | --- | --- | --- |
| SS1400 | GCG CCCGGG GCCTAATTGAGAGAAGTTTCTATAGAA | amplify *aad9* | *XmaI* |
| SS1404 | GAT CGGCCG ATCTGCA | add EagI site at PstI | *EagI* |
| SS1408 | AATTGAT CGGCCG ATC | add EagI site at EcoRI | *EagI* |
| SS1429 | GCG GAATTC CGGCCG GGATCGATCTGTATAATAAAGAATAATTA | amplify *aad9* | *EcoRI, EagI* |
| SS1430 | GCG GAATTC CGGCCG GCCTAATTGAGAGAAGTTTCTATAGAA | amplify *aad9* | *EcoRI, EagI* |
| SS1454 | CGC GGTACC CGGCCAGCCTCGCAGAGCAGGATT | amplify *oriT* | *KpnI* |
| SS1455 | CGC GGTACC GGATCGCGCTTTTCCGCTGCATA | amplify *oriT* | *KpnI* |
| RP52 | ATA CTGCAG CCTAGG TT GGATCC GGCCACATAGATGGCGTCGCT | amplify *aphA*(3) | *PstI, AvrII, BamHI* |
| RP53 | ATA CTGCAG CGGCCGC AA TCTAGA CTAAAACAATTCATCCAGTA | amplify *aphA*(3) | *PstI, NotI, XbaI* |
| RP58 | CTAGATT GCGGCCGC AGACCGGGGACTTATCAGCCAACCTGT | insert IR | *NotI* |
| RP59 | GGCCACAGGTTGGCTGATAAGTCCCCGGTCT GCGGCCGC AAT | insert IR | *NotI* |
| RP68 | CGC GGTCTC AGATCT GGAGAATACGGCGGGTGG | delete BamHI site | *BsaI, BglII* |
| RP69 | CGC GGTCTC CCCGGG GAACCTACACTTGCTGC | delete BamHI site | *BsaI, XmaI* |
| RP193 | TAT GGTCTC ACTAGGTT ACGCGT GAAGATCAGCAGTTCAACCTGTTGATAGTACGTACTAAGCTCTC | amplify R6Kγ *ori* and enhancer | *BsaI, MluI* |
| RP270 | TAT GGTCTC ACGCGCCATGTCAGCCGTTAAGTGTTCCTG | amplify R6Kγ *ori* and enhancer | *BsaI* |
| RP378 | TGATGGTCGTCATCTACCTG | amplify transposon features from pRP1269 | none |
| RP426 | CGC GGTCTC ACCGGCTCTAGTTAATGTGTAACGTAAC | amplify *bgaB* | *BsaI* |
| RP462 | TAT GGTCTC AAGCTAAA CGGCCG AA CCCGGG CGCCATGTCAGCCGTTAAGTGTTCCTG | amplify transposon features from pRP1116 | *BsaI, EagI, XmaI* |
| RP463 | TAT GGTCTC ACGCGAGGTTTAAATCCTTATCGTTATGGG | amplify transposon features from pRP1116 | *BsaI* |
| RP473 | TTGGTTCCCGCTTTTATGTG | amplify transposon features from pRP1269 | none |
| RP490 | TAT GGTCTC AGGCCGTA GGTACC AGCTGTTGACAATTAATCATCC | amplify *aad9* | BsaI, KpnI |
| RP491 | GGCCACAGGTTGGCTGATAAGTCCCCGGTCT CGGCCG TA GAATTC ATGGTAC | insert IR | EagI, EcoRI |
| RP492 | CAT GAATTC TA CGGCCG AGACCGGGGACTTATCAGCCAACCTGT | insert IR | EcoRI, EagI |
| RP495 | CGC GGTCTC ACCGGCTTAGCATATTATGTTGCCAACTGTC | amplify *bgaB* | BsaI |
| RP540 | TAT GGTCTC CCGCGTAA CCTAGG AAGACCGGGGACTTATCA TCCAAC CTGTC | modify IR to include MmeI site | BsaI, AvrII, MmeI |
| RP541 | GTCGGTTTTCG GGATCC ATATGACG | modify IR to include MmeI site | BamHI |
